# Recovery of the gut microbiome following enteric infection and persistence of antimicrobial resistance genes in specific microbial hosts

**DOI:** 10.1038/s41598-023-42822-7

**Published:** 2023-09-19

**Authors:** Zoe A. Hansen, Karla Vasco, James T. Rudrik, Kim T. Scribner, Lixin Zhang, Shannon D. Manning

**Affiliations:** 1 Departments of Microbiology and Molecular Genetics, Michigan State University, East Lansing, MI 48824 USA; 2 Bureau of Laboratories, The Michigan Department of Health and Human Services, Lansing, MI 48906 USA; 3 Fisheries and Wildlife, Michigan State University, East Lansing, MI 48824 USA; 4 Epidemiology and Biostatistics, Michigan State University, East Lansing, MI 48824 USA

**Keywords:** Clinical microbiology, Metagenomics, Microbiome, Infectious diseases

## Abstract

Enteric pathogens cause widespread foodborne illness and are increasingly resistant to important antibiotics yet their ecological impact on the gut microbiome and resistome is not fully understood. Herein, shotgun metagenome sequencing was applied to stool DNA from 60 patients (cases) during an enteric bacterial infection and after recovery (follow-ups). Overall, the case samples harbored more antimicrobial resistance genes (ARGs) with greater resistome diversity than the follow-up samples (*p* < 0.001), while follow-ups had more diverse gut microbiota (*p* < 0.001). Although cases were primarily defined by genera *Escherichia*, *Salmonella,* and *Shigella* along with ARGs for multi-compound and multidrug resistance, follow-ups had a greater abundance of Bacteroidetes and Firmicutes phyla and resistance genes for tetracyclines, macrolides, lincosamides, and streptogramins, and aminoglycosides. A host-tracking analysis revealed that *Escherichia* was the primary bacterial host of ARGs in both cases and follow-ups, with a greater abundance occurring during infection. Eleven distinct extended spectrum beta-lactamase (ESBL) genes were identified during infection, with some detectable upon recovery, highlighting the potential for gene transfer within the community. Because of the increasing incidence of disease caused by foodborne pathogens and their role in harboring and transferring resistance determinants, this study enhances our understanding of how enteric infections impact human gut ecology.

## Introduction

Foodborne illness caused by enteric pathogens impacts ~ 9.4 million people in the United States each year, with over one-third being attributed to bacterial pathogens^1^. In 2019, the Centers for Disease Control and Prevention (CDC) documented a marked increase in the incidence of foodborne infection caused by *Campylobacter* and Shiga toxin-producing *Escherichia coli* (STEC)^2^. *Salmonella* and *Shigella* also contribute to a high incidence of infections, though case numbers remained unchanged relative to previous years^2^. In addition to their role in enteric disease, *Campylobacter*, non-Typhoidal *Salmonella*, *Shigella*, and members of *Enterobacteriaceae* (e.g., *Escherichia*) have been classified by the CDC as serious threats for harboring and transmitting antimicrobial resistance^2^. Indeed, each of these pathogens have been shown to transfer ARGs horizontally within and between microbial species residing in a niche^3^. Such resistance determinants can cross environmental boundaries, thereby increasing frequencies of ARGs or mobile elements within different hosts and environments and enhancing the likelihood of horizontal gene transfer (HGT).

The consequences of enteric infection on the health of the human gut microbiome are not fully understood. A prior study conducted in our lab showed a marked decrease in gut microbiota diversity attributed to enteric infection as determined using 16S rRNA sequencing^4^. This lack of diversity was suggested to reduce beneficial microbially-mediated metabolism and exacerbate gut inflammation^5^. Similarly, other studies have demonstrated an increase in the proportion of Proteobacteria upon infection with *Salmonella*¸ *Campylobacter*, *Shigella*, and other pathogens in multiple host organisms^6^. More recently, we documented differences in the gut resistome, or compilation of antimicrobial resistance genes (ARGs), in patients with *Campylobacter* infections when compared to their healthy family members^7^. The potential ecological repercussions relevant to recovery from enteric infection, however, have yet to be explored using shotgun metagenome sequencing. If the microbiome demonstrates a certain degree of resilience, then the magnitude of the perturbations should not be large, and microbiota composition should recover over time^8^. In the context of pathogen invasion, various ecological interactions such as direct antagonism from commensal microbes, resource competition and competitive exclusion, and secondary metabolite production, must be considered^9,10^. Each of these factors may influence the success of an enteric pathogen in the gut environment as well as the time it takes to restore the affected microbiome to a healthy state, allowing the patient to recover from the acute infection.

Consideration must also be given to the invading pathogen, which can potentially introduce virulence and antimicrobial resistance determinants into the gut community. Indeed, pathogens harboring ARGs can transfer these to other gut microbes during infection or vice versa, thereby transforming the gut into a resistance gene reservoir^11^. This reservoir is particularly concerning given that pathobionts residing in the community can acquire genetic factors that encode for virulence properties as well as resistance to clinically important antibiotics.

To understand how infection by and recovery from enteric pathogens influences the human gut resistome and microbiome, we applied shotgun metagenome sequencing to stool-derived DNA from 60 patients collected during their infection and again after they recovered from the infection. Because our prior 16S sequencing analysis showed that infection with enteric pathogens altered the relative abundance of specific microbial populations in the gut^4^, we hypothesized that ARGs harbored by microbes that “bloom” during infection will also increase in abundance. Use of a novel sequence-based approach enabled the identification of bacterial hosts harboring specific ARGs^12^. This approach has yet to be applied to enteric infections and can advance understanding of how drug resistance spreads and is maintained within a dysbiotic gut microbiome and is maintained in a healthy gut microbiome. Further defining the impacts of these infections on the composition and function of the gut microbiome is necessary to counteract the dissemination of drug resistance and guide the discovery of novel therapeutic solutions such as fecal microbiome transplantation or bacteriophage therapies.

## Methods

### Sample collection and sequencing

Sixty stools were obtained from patients with enteric infections (cases) caused by *Campylobacter* (n = 24), *Salmonella* (n = 29) *Shigella* (n = 4), and Shiga toxin-producing *E. coli* (STEC) (n = 3) from 2011–2015. Stools were preserved in Cary-Blair transport media and submitted to the Michigan Department of Health and Human Services (MDHHS) in collaboration with four hospitals as described^4^. Patient demographics, exposures, and symptoms were reported through the Michigan Disease Surveillance System (MDSS). Counties were classified as ‘rural’ or ‘urban’ as was done in our prior analysis^7^. Each patient submitted a follow-up sample 1 week to 29 weeks after their acute infection, yielding 120 paired samples for analysis; these are referred to as “follow-ups” for simplicity. Moreover, 91 stools from household members were included as controls and 38 of these controls were from the same household as the 60 patients sampled during and after infection. Control samples were submitted 5–29 weeks after the cases’ infection. Resistome data from *Campylobacter* patients were examined previously^7^, though no prior metagenome analyses were performed on the post-recovery samples. Study protocols and consent procedures were performed in accordance with the relevant guidelines and regulations set by the Declaration of Helsinki. Informed consent was obtained from participants and/or their legal guardians prior to enrollment. All data were stripped of personal identifying information. Final approval to conduct the study was granted by the Institutional Review Boards at MSU (IRB #10-736SM), the MDHHS (842-PHALAB), and the four participating hospital laboratories as described in our prior study^4^.

Metagenomic DNA was extracted, sheared, and normalized as described previously^4^. Libraries were constructed using the TruSeq Nano library kit (Illumina, Inc., San Diego, CA, USA) and shotgun sequencing was conducted in four runs (batches) using an Illumina HiSeq 2500; the samples were not selected for each batch in a specific order. Base calling was performed using Real Time Analysis (RTA) v1.18.66 (Illumina) software, while the output was demultiplexed to separate the reads from each sample and converted to FastQ files with Bcl2fastq v2 + .

### Reads-based identification of antimicrobial resistance genes (ARGs)

The AmrPlusPlus v2.0 pipeline was used for quality control checking, aligning, and annotating metagenomic fragments with the MEGARes 2.0 database^13^ using previously described parameters^7^. Reads were mapped to the human genome, GRCh38 (GRCh38_latest_genomic.fna.gz, downloaded December 2020), in RefSeq using the Burrows-Wheeler Aligner (BWA)^14^ and removed using SAMTools^15^ and BEDTools^16^. The non-host FASTQ files were stored and aligned to MEGARes 2.0^13^ to identify ARGs using default values. These aligned non-host reads were deduplicated and annotated with the ResistomeAnalyzer tool (identity threshold of ≥ 80%) in AmrPlusPlus v2.0 to quantify ARG abundance per sample; the RarefactionAnalzyer tool estimated sequencing depth. Following annotation and quantification of ARG abundances, MicrobeCensus^17^ was used to determine the average genome size (AGS) and number of genome equivalents (GE) for normalizing ARG and taxonomic abundances. Because we were most interested in identifying differences in gene occurrence between samples, we used GE for normalization. These were determined by dividing the total library size (i.e., number of base pairs) by the AGS within a sample as described in our studies of the gut microbiome in *Campylobacter* cases and their healthy family members^7^ and cattle following antibiotic treatment^18^.

### Reads-based classification of microbial taxa

Non-host paired-end reads were taxonomically annotated with Kaiju (version 1.7.4), a protein-based classifier that translates reads to amino acid sequences while searching for maximum exact matches (MEMs) among microbial reference genomes^19^. The National Center for Biotechnology Information (NCBI) BLAST *nr* reference database was used with previously published parameters^7^. Raw abundances of reads assigned to taxa were normalized by the estimated number of GEs. Those sequencing reads without enough resolution were categorized as “unassigned”, which comprised on average ≥ 50% of annotated reads at the genus and species levels and 15% at the phylum level. Variation in the percentage of unassigned read was observed across samples and is dependent on the sequencing depth and annotation quality. Consequently, analyses were performed at the phylum and genus levels and composition analysis was restricted to assigned reads.

### Assembly-based identification of ARGs

Non-host FASTQ files were also used for metagenome assembly after employing BBTools for paired end read merging using the ‘bbmerge-auto.sh’ script (https://sourceforge.net/projects/bbmap/); reads that failed merging were error-corrected using Tadpole and reexamined. If merging continued to fail, reads were extended 20 bp and merging was iterated up to five additional times or unmerged reads were included. Assembly was performed with MEGAHIT^20^ using the merged and paired-end reads. The Quality Assessment Tool for Genome Assemblies (QUAST)^21^ evaluated assembly quality and coverage.

In addition, anvi’o was used to analyze microbial genomes from metagenomes as described^22^. Briefly, assembled contigs were reformatted using ‘anvi-script-reformat-fasta’ to generate a contigs database per sample with ‘anvi-gen-contigs-database’. The script ‘anvi-run-hmms’ was used to populate the contigs database with hits detected using Hidden Markov Models, which improves assembly annotation. Prodigal^23^ was used in the script ‘anvi-get-sequences-for-gene-calls’ to obtain amino acid sequences of genes present in the assemblies; these gene calls were then used in the ARG-carrying contigs (ACC) analysis.

### Identifying bacterial hosts harboring ARGs

Gene calls from anvi’o were used to identify ARG-carrying contigs (ACCs) by aligning amino acid sequences to the HMD-ARG database^24^ using DIAMOND^25^ with a modified pipeline that was described previously^12,26^. Significantly more contigs were found in the follow-up samples relative to the case samples; the former samples also had a greater total length of contigs (Figure S3). SAM files were filtered to identify contigs with ARG hits, and Seqtk (https://github.com/lh3/seqtk) was used to select these ACCs for subsequent alignment to the BLAST database v5.0 using blastp. To reduce spurious annotations, an E-value of 0.00001 cutoff was used with a maximum of 50 target sequences (i.e., 50 matches per contig). This value indicates that there is only a 1 in 100,000 chance that a sequence alignment would occur by chance; only the top-50 taxonomic matches (as sorted by E-value) were used. One *Campylobacter* sample could not be annotated and was excluded along with the paired follow-up sample leaving 59 pairs (118 samples) for analysis.

Alignment output was used to identify taxa associated with each ARG on a contig. Since 50 matches were allowed per contig, a custom Python script (‘ERIN_ACCpipeline_blastp_ merge’) was used to quantify the average proportion of each genus per sample on the ACCs and the average percentage of different ARGs per genus within all ACCs in a sample. Taxa with the most hits per contig were considered the most likely to harbor a given ARG. The average percent identity for taxonomic annotations across all ACCs in each sample ranged from 86.6% to 99.8% per sample with an overall average of 93.6%.

### Abundance and diversity analyses

The identity and diversity of ARGs and taxa were determined for all samples. For the resistome analyses, the gene, group, mechanism, class, and type levels were used^13^. Actual estimated abundance of ARGs and taxa was determined by normalizing raw abundance counts to the number of GEs per sample. Relative abundance was calculated by dividing the number of GE-normalized reads assigned to a specific feature by the total number of GE-normalized reads for that sample. Alpha diversity metrics such as richness, Shannon diversity, and Pielou’s evenness score were estimated using the vegan package^27^ in R (https://www.R-project.org/). Nonparametric tests evaluated differences between groups and the Shapiro–Wilk test indicated that both the resistome and microbiota data were not normally distributed (Table S1).

The Wilcoxon signed-rank test was used to detect significant differences between paired samples, whereas the Wilcoxon rank-sum test was applied to unpaired samples. Beta diversity metrics and ordination plots (e.g., Principal Coordinate Analysis (PCoA)) based on Bray–Curtis dissimilarity at the gene and group (ARGs) or species and genus (taxa) levels were also estimated with vegan^27^. The overall mean dissimilarity among cases and follow-ups was compared to the mean dissimilarity between paired samples using a Welch’s t-test (Figure S1). A Permutational Analysis of Variance (PERMANOVA) was calculated using the Bray–Curtis dissimilarities in R to assess differences in centroids (mean) between cases and follow-ups for both the resistome and microbiota composition; Permutational Analysis of Multivariate Dispersion (PERMDISP) detected differences in dispersion (degree of spread) of these groups.

### Differential abundance and continuous structure analysis of taxa and ARGs

To assess representative features in cases and follow-ups, MMUPHin was used to construct general linear models relating sample features to relative abundances^28^. Batch adjustment of relative abundance data was performed by sequencing run, which significantly influenced the distribution of points in the microbiota ordination (Figure S2). To identify differentially abundant ARGs and taxa, a linear model was constructed with follow-ups serving as the reference for the fixed effect. Age in years, average genome size, number of GEs, year of collection, and use of antibiotics were included as covariates. Significance values were adjusted using the Benjamini–Hochberg method of correction for multiple hypothesis testing (q-value representing False Discovery Rate). The Analysis of Compositions of Microbiomes with Bias Correction (ANCOM-BC) method^29^, which considers absolute abundances from the GE-normalized counts as input but cannot implement a mixed model with fixed and random effects, was used for differential abundance testing. Results produced by ANCOM-BC were concordant with those generated by MMUPHin at each comparison level, though differences in rank of correlation were observed for some features.

MMUPHin^28^ was subsequently used to identify taxonomic or resistance gene tradeoffs that impact data structure in ordination. The ‘continuous_discover()’ function was applied to relative abundance data, which performs unsupervised continuous structure discovery using Principal Components Analysis (PCA). Continuous structure scores (“loadings”) comprising the top components were compared across batches to identify “consensus” loadings assigned to microbial features. The ‘var_perc_cutoff()’ parameter, which filters out the top components accounting for a set proportion of the variability within the samples, was set to 0.75 for phylum and ARG class levels, 0.50 for genus and ARG groups, and 0.40 for species. Plots were constructed to visualize drivers of continuous data structure and to overlay data onto ordination plots based on Bray–Curtis dissimilarity of microbiota or resistome relative abundances.

## Results

### Study population

Among the 60 cases, 28 were male (46.7%) and 32 were female (53.3%) ranging between 1.5 and 90 years of age; most patients were between 19 and 64 years (n = 26; 43.3%) or less than 9 years (n = 16; 26.7%). No difference in the proportion of stool submissions was observed by year, though the fewest (n = 13.3%) were recovered in 2011 and the most (36.7%) in 2013. Among the 59 patients reporting symptoms, 50 (84.8%) had abdominal pain, 57 (96.6%) had diarrhea, and 22 (37.3%) reported blood in the stool. Seventeen (28.3%) cases required hospitalization and 33 (55.0%) resided in a rural area. Most cases did not take antibiotics within two weeks of sampling, though five (8.3%) reported use of amoxicillin (n = 2), azithromycin (n = 1), ciprofloxacin (n = 1), or an unknown antibiotic (n = 1) before submitting the follow-up sample.

Most follow-up samples were collected 51–100 days (n = 20; 33.9%) or 101–150 days (n = 28; 47.5%) post-infection, however, a small number was submitted ≤ 50 (n = 4; 6.78%) or > 150 (n = 7; 11.9%) days after the initial sample was collected; the date was missing for one patient. The range of follow-up submissions was 8 to 205 days post-recovery with an average of 107.9 days. Similar to the cases, five controls reported antibiotic use within 2 weeks prior to sample collection for unknown reasons. Antibiotics taken included amoxicillin (n = 2), azithromycin (n = 1), and ciprofloxacin (n = 1); one respondent did not report the drug class.

### Metagenome sequencing and assembly metrics

Sequencing of the stool DNA from 60 patients during and after infection (cases = 60; follow-ups = 60) and 91 household controls (total = 211) resulted in a total yield of 285.4 Gbp and 678.7 million paired reads (PF) across samples (Table 1). Each sample yielded an average of 1.4 Gbp of data with an average Q-score of 34.81 across both forward and reverse reads; a score of Q30 designates a 99.9% accuracy in sequencing calls. Quality trimming with Trimmomatic resulted in an average of 3.18 million 150 bp surviving paired-end reads; on average, 14,978 reads were dropped from samples due to poor quality. There were no significant differences for surviving paired-end reads between cases and follow-ups (n_case_ = 3.15e6 vs. n_follow-up_ = 3.04e6; *p* = 0.53), cases and controls (n_control_ = 3.29e6; *p* = 0.11) and controls and follow-ups (*p* = 0.061) (Figure S3).

**Table 1:** Metagenomic sequencing metrics from fecal DNA collected from patients during (Case) and after (Follow-up) acute enteric infection, and household controls (Control).

Metric	Sample	Value
Average yield (Gbp)	Case	1.388153
	Follow-up	1.347647
	Control	1.438958
PF reads	Case	3,166,668
	Follow-up	3,048,540
	Control	3,360,604
Average Q score	Case	35.17488
	Follow-up	35.57133
	Control	34.09067
Surviving reads (after trimming)	Case	3,152,079
	Follow-up	3,037,234
	Control	3,293,993
Non-host reads	Case	3,946,049
	Follow-up	4,428,700
	Control	5,143,197
Average genome size (AGS)	Case	4,309,526
	Follow-up	4,155,749
	Control	3,912,608
Genome equivalents	Case	204.7146
	Follow-up	240.2219
	Control	282.3293
Number of contigs	Case	30,654
	Follow-up	47,900
	Control	57,031
Total contig length	Case	57,957,505
	Follow-up	85,358,266
	Control	94,713,414
N50	Case	9485
	Follow-up	4267
	Control	3248
Average coverage depth	Case	17.6
	Follow-up	10.9
	Control	10.1

On average, there were 4.6 million non-human paired-end reads per sample; however, this metric ranged from 195, 929 to 13.9 million. The prevalence of non-host reads was somewhat sample-specific and the number of host and non-host reads differed significantly between case, follow-up, and control samples (Figure S4). Controls had more non-host reads than cases (n_control_ = 5.14e6 vs. n_case_ = 3.95e6; *p* = 2.4e−6) and follow-ups (n_follow-up_ = 4.43e6; *p* = 0.0039), while follow-ups had more non-host reads than cases (*p* = 0.04). All non-host reads were used to estimate metagenomic coverage with Nonpareil^30^. Mean coverage across cases, follow-ups and controls was 84.2%; among cases and follow-ups alone, coverage was 86.3% (Figure S5).

The AGS across all samples was 4.09 million bp with cases having significantly larger sizes than controls (AGS_case_ = 4,309,526 vs. AGS_control_ = 3,912,608; *p* = 2.2e−4). There was no significant difference in AGS between cases and follow-ups (AGS_follow-ups_ = 4,155,749; *p* = 0.086) or follow-ups and controls (p = 0.086). The average number of GE among all samples was 248.29, with controls registering significantly more than cases (GE_control_ = 282 vs. GE _case_ = 205; *p* = 2.4e−4) and follow-ups (GE_follow-up_ = 240; *p* = 0.035). Follow-ups had significantly more GE than cases as well (*p* = 0.029) (Figure S6).

Metagenome assembly was used to assess ARG-carrying contigs (ACCs) among microbiota members within the samples (Figure S7). Cases had significantly fewer assembled contigs than the follow-up (N_case_ = 30,654 vs. N_follow-up_ = 47,900; *p* = 4.9e−5) and control samples (N_control_ = 57,031; *p* = 1.0e−7) and registered shorter contig lengths overall (L_case_ = 57.96 Mbp vs. L_follow-up_ = 85.36 Mbp, *p* = 2.2e−4; L_control_ = 94.71 Mb, *p* = 1.5e−5). Control and follow-up samples did not differ in the number of contigs (*p* = 0.069) or contig length (*p* = 0.28). The N50 value, which designates the length at which half of the total assembly length is contained in contigs of that size or larger, registered an average of 9485 bp in cases, 4267 in follow-ups, and 3248 in controls (N50_case_ vs. N50_follow-up_, *p* = 0.0363; N50_case_ vs. N50_control_, *p* = 1.5e−5; N50_control_ vs. N50_follow-up_, *p* = 0.0042). The average coverage depth for assemblies was significantly greater among cases than follow-ups (D_case_ = 17.6 vs. D_follow-up_ = 10.9; *p* = 0.011) and controls (D_control_ = 10.1; *p* = 6.3e−4), which may be due to cases having both smaller and fewer contigs overall. Coverage depth did not differ between follow-up and control samples (*p* = 0.38).

### Changes in resistome composition and diversity post-recovery

**Figure 1:**
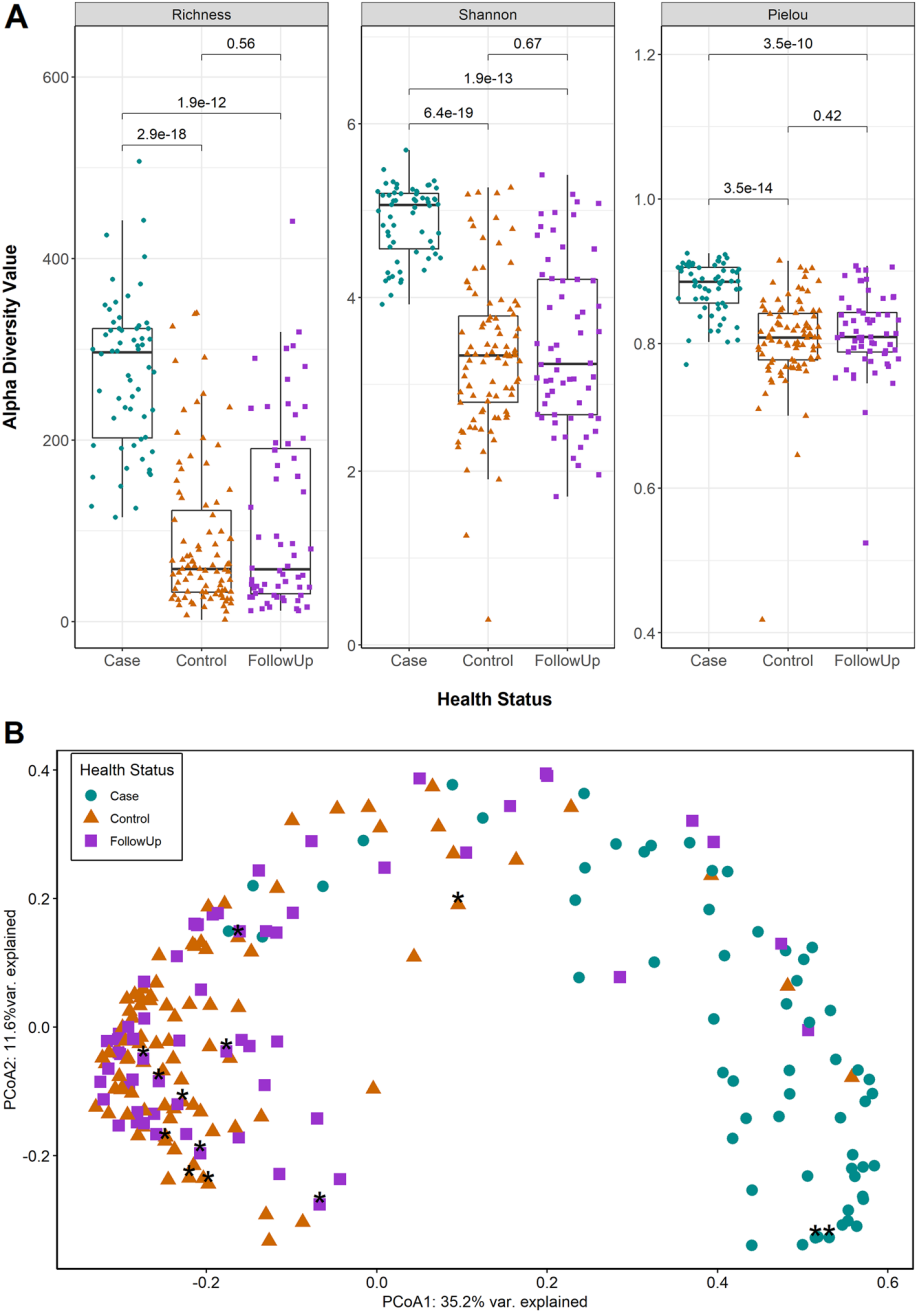
Resistome diversity and composition differ significantly during infection and after recovery. (**A**) Three alpha diversity measures (Richness, Shannon’s Diversity Index, and Pielou’s Evenness Index) are presented. Case samples (Case) are indicated with green dots and follow-up samples (FollowUp) are purple squares; household control samples (Control) are shown as orange triangles. Points are slightly offset from the vertical to allow interpretation of all samples. The median of each measure is indicated by the thick bar within each box and the first and third quartiles are indicated at the bottom and top of the box, respectively. Adjusted *P*-values were calculated using the Wilcoxon signed-rank test for paired samples and are shown above the comparison bar within each plot. (**B**) A Principal Coordinates Analysis (PCoA) plot of case (green circles), follow-up (purple squares), and control (orange triangles) resistomes based on Bray–Curtis dissimilarity calculated from gene-level abundances. The first and second coordinates include the corresponding percentage of similarity explained. Patients that used antibiotics two weeks prior to sample collection are indicated by black asterisks.

Among the 120 stool samples from cases and follow-ups, 1,338 ARGs were identified encoding resistance to biocides, antibiotic drugs, metals, and multi-compound substrates comprising 474 distinct gene groups or operons. These genes represented 120 distinct mechanisms conferring resistance to 44 classes of compounds. In all, the case samples had a significantly greater mean ARG richness than follow-up or control samples (S_cases_ = 274 vs. S_follow-ups_ = 111; *p* = 1.9e−12; S_cases_ vs. S_controls_ = 90.2; *p* = 2.9e−18) (Fig. 1A). The Shannon Diversity Index for ARGs was also greater in cases than follow-ups (H_cases_ = 4.79 vs. H_follow-ups_ = 3.36; *p* = 1.9e−13) and controls (H_controls_ = 3.34; *p* = 6.4e−19). The Pielou’s evenness index followed a similar trend in which cases were significantly greater than follow-ups (J’_cases_ = 0.87 vs. J’_follow-ups_ = 0.80; *p* = 3.5e−10) and controls (J’_controls_ = 0.805; *p* = 3.5e−14). Notably, follow-up samples did not significantly differ from their family member controls in ARG diversity, suggesting recovery to a “normal” ARG level of post-infection.

The resistome composition among cases, follow-ups, and controls also differed as was demonstrated in a PCoA based on the Bray–Curtis dissimilarity (PERMANOVA *p* = 0.000999; F = 32.40) (Fig. 1B), though no difference was observed in the level of dispersion between groups (PERMDISP *p* = 0.511; F = 0.732). The samples from cases reporting antibiotic use did not cluster separately from those without antibiotics. In the subset of data representing only the case-follow-up pairs, residence type, antibiotic use, gender, age, hospital, county of origin, stool type, sequencing run, and number of days between samplings were fit to the corresponding ordination; age in years (*p* = 0.013) and year of collection (*p* = 0.043) independently influenced the distribution of points. Residence location, hospital, and the number of days since infection, however, only trended toward significance. Moreover, antibiotic use and the pathogen responsible for the acute infections did not have significant effects on alpha or beta diversity trends, at least for patients with *Campylobacter* and *Salmonella* infections (Figure S8). Too few patients had *Shigella* and STEC infections, thereby preventing comparisons with these samples.

### Changes in gut microbiota composition and diversity post-recovery

**Figure 2:**
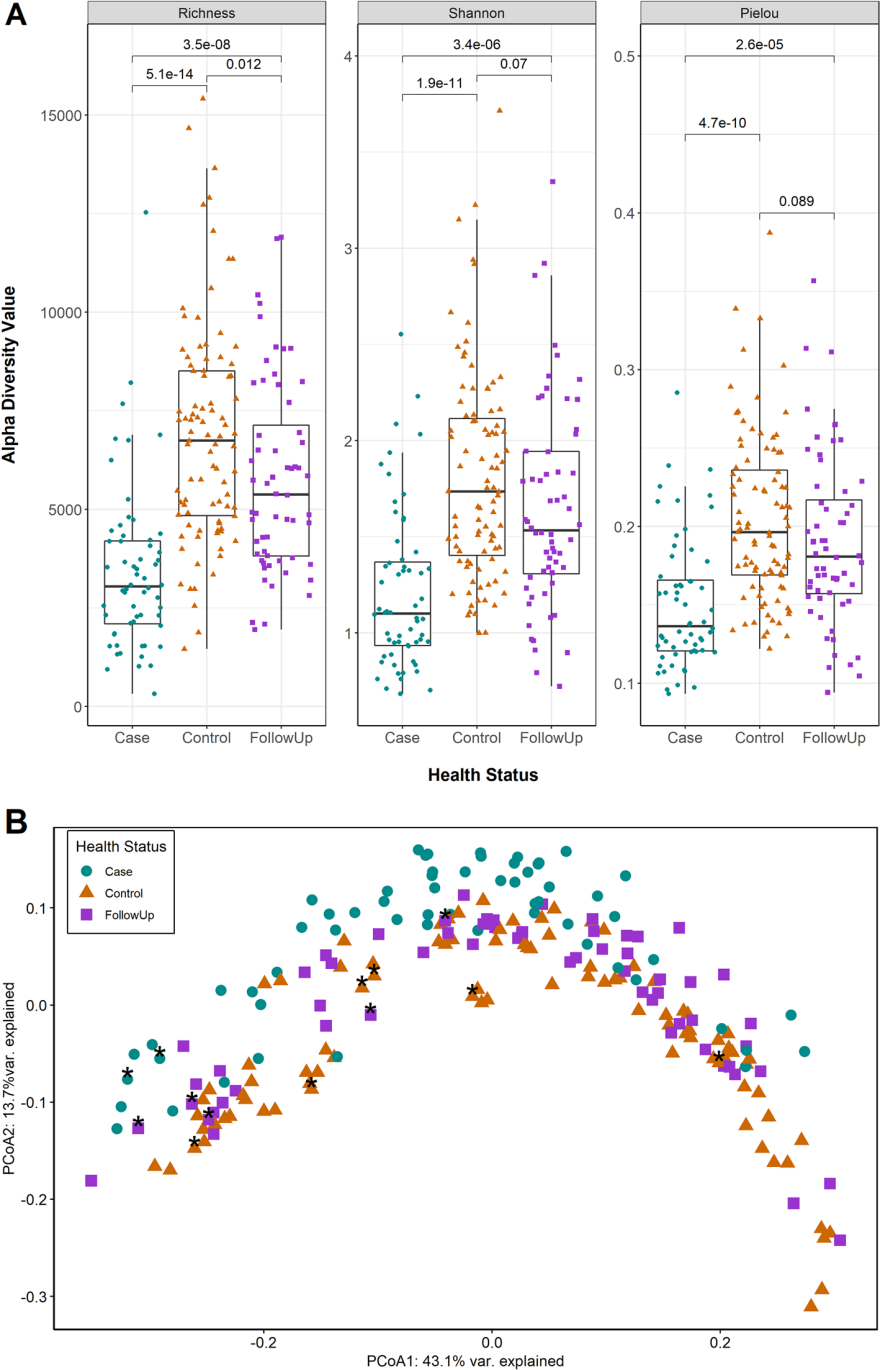
Gut microbiota diversity is greater after recovery and compositional differences between samples are nuanced. (**A**) Box plots show the microbiome alpha diversity measures (Pielou’s Evenness Index, Richness, Shannon Diversity Index). Separate points represent case (Case, green), follow-up (FollowUp, purple), and control (Control, orange) samples and are offset from the vertical for clarity. The median is indicated by the thick black bar, while the first and third quartiles are represented by lines at the bottom and top of the box, respectively. Adjusted P-values were calculated using the Wilcoxon signed-rank test for paired samples and are shown above the comparison bars. (**B**) A Principal Coordinates Analysis plot is shown for case (Case, green circles), follow-up (FollowUp, purple squares), and control (Control, orange triangles) microbiota based on Bray–Curtis dissimilarity at the species level. The first and second coordinate are shown and include the corresponding percentage of similarity explained. Samples from individuals self-reporting use of antibiotics two weeks prior to sample collection are indicated by black asterisks.

The gut microbiota were more diverse in the follow-up and control samples than the case samples (Fig. 2A) with a significantly greater mean species richness (S_cases_ = 3,426, S_follow-ups_ = 5,789; *p* = 3.5e−08; S_cases_ vs. S_controls_ = 6,872; *p* = 5.1e−14), mean evenness (J’_case_ = 0.150, J’_follow-up_ = 0.190; *p* = 9.8e−06; J’_case_ vs. J’_control_ = 0.205; *p* = 4.7e−10), and Shannon Diversity (H_cases_ = 1.21, H_follow-ups_ = 1.65; *p* = 1.3e−06; H_cases_ vs. H_controls_ = 1.81; *p* = 1.9e−11). When compared to healthy (control) samples from members of the same household, the follow-up samples were similar for Shannon Diversity and evenness despite showing differences in microbiota richness (S_follow-ups_ = 5789, S_controls_ = 6872; *p* = 0.012, Wilcoxon rank-sum test (two-sided, unpaired)).

The microbiota composition was significantly different between the case, follow-up, and control samples (PERMANOVA *p* = 0.000999, F = 6.75; Fig. 2B). However, a difference in the dispersion of points between groups (PERMDISP *p* = 0.025; F = 3.42) was also observed, which was driven by a discrepancy in spread between cases and controls (*p* = 0.027; Tukey’s Honest Significant Difference Test). Age (*p* = 0.008), sequencing run (*p* = 0.001), average genome size (*p* = 0.001), number of genome equivalents (*p* = 0.001), year of sampling (*p* = 0.005), days to follow-up (*p* = 0.013), hospital (*p* = 0.030), and antibiotic use (*p* = 0.008) significantly impacted the point distribution of paired cases and follow-ups (Figure S9). No differences were observed by the causative agent, at least for those cases with *Campylobacter* and *Salmonella* infections that had adequate sample sizes for analysis (Figure S10).

### ARG composition and abundance varied during and after infection

**Figure 3:**
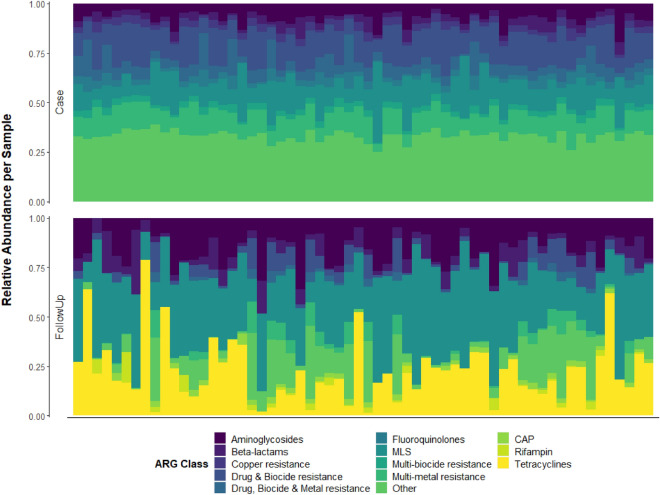
Relative abundance of the top-10 resistance gene classes differs between case and follow-up samples. The relative abundance of resistance genes assigned to the top-10 most abundant compound classes is shown for cases (Case, top panel) and follow-ups (FollowUp, bottom panel). Each column represents the resistome from one individual and columns are ordered by the paired samples, meaning that each set of two columns refers to the same individual during or after infection. Relative abundances were determined using raw gene abundances normalized by the approximate number of genome equivalents in the sample as determined using MicrobeCensus ^17^. CAP = cationic antimicrobial peptides; MLS = Macrolide, Lincosamide, Streptogramin; MDR = Multidrug resistance; QACs = Quaternary Ammonium Compounds.

The relative abundance of ARGs differed between groups (Figure S11). However, because the ARG profiles did not differ between household controls and follow-ups, we limited the in-depth ARG analyses to the case and follow-up pairs. Among these samples, the top-three resistance classes in cases accounted for 39.8% of the total resistance genes relative to 71.0% for follow-ups, supporting the observation of greater resistome diversity during infection. Classes for drugs and biocides (15.1%), MLS (macrolides, lincosamides, and streptogramins) (13.3%), and multi-metals (11.3%) were most abundant in cases compared to MLS (33.5%), tetracyclines (22.0%), and aminoglycosides (15.5%) in the follow-ups (Fig. 3). Differential abundance analysis revealed that classes for multi-metal resistance (coef = − 0.243; q-value = 1.04e−04), drug and biocide resistance genes (coef = − 0.243; q-value = 1.46e−03), drug, metal, biocide resistance (coef = − 0.212; q-value = 7.86e−09), and fluoroquinolone resistance genes (coef = -0.168; q-value = 8.19e−10) were more abundant in cases (Figure S12). Comparatively, tetracycline resistance genes (coef = 0.352; q-value = 2.26e−05) were more abundant in the follow-up samples followed by MLS (coef = 0.251; q-value = 1.49e−25) and aminoglycoside (coef = 0.118; q-value = 7.86e−09) genes, a result that is concordant with the resistomes of healthy controls analyzed in our prior study^7^.

At the group level, specific ARGs were identified for the predominant classes. In the cases, the most abundant groups were MLS23S (11.9%) conferring MLS resistance, *rpoB* (2.8%), a rifampin resistance gene, and A16S (3.8%), which is important for aminoglycoside resistance (Figure S13). Moreover, the differential abundance analysis detected the MDR genes *rpoB* (coef = − 0.123; q-value = 6.30e−05) and *mdtC* (coef = − 0.103; q-value = 4.97e−09) to be the most differentiating ARG groups for cases (Figure S14). Genes such as *parC* (coef = − 0.102; q-value = 3.90e−11) and *gyrA* (coef = − 0.101; q-value = 7.38e−08), which encode resistance to fluoroquinolones, were also more abundant in cases.

In the follow-ups, the most abundant groups were for MLS, tetracycline, and aminoglycoside resistance, with MLS23S (n = 6.6; 24.3%), *tetQ* (n = 4.0; 17.0%), A16S (n = 2.4; 9.5%), and *cfx* (n = 0.84; 3.8%) predominating, respectively (Figure S13). *tetQ* had the greatest differential abundance in favor of follow-ups (coef = 0.30; q-value = 6.56e−05), a finding confirmed by continuous structure analysis (Figure S15). Despite its noted prevalence among cases, MLS23S was also a defining group for follow-ups since it comprised a greater proportion of ARGs (coef = 0.172; q-value = 5.54e−06) (Figure S14). The *cfx* (coef = 0.124; q-value = 0.0078) and other genes important for MLS resistance such as *mefE* (coef = 0.08; q-value = 3.54e−07) and *ermF* (coef = 0.07; q-value = 3.68e−08), were also more abundant in the follow-ups as were aminoglycoside resistance genes *ant(6)* (coef = 0.103; q-value = 5.23e−04) and A16S (coef = 0.092; q-value = 5.14e−04). Notably, both *tetQ* and *cfx* were also highly abundant among healthy controls in a hierarchical clustering analysis that we performed previously when exploring the impacts of *Campylobacter* infection on the gut resistome^7^.

### Taxa composition and abundance differ markedly during and after infection

Similar to our ARG analysis, controls were excluded due to notable overlap with the follow-ups and our desire to better understand the trajectory of microbiome recovery following infection. Both cases and follow-ups were dominated by Bacteria (relative abundance = 82.0% and 84.4%, respectively) with fewer Archaea or Eukarya, a disparity that may be due to less successful extraction of DNA from these groups. During infection, cases had a high proportion of Proteobacteria (37.1%), which was also confirmed in the differential abundance analysis (coef = − 0.461; q-value = 9.35e−28), and a decreased abundance of Bacteroidetes (29.6%) and Firmicutes (13.7%) (Figure S16). Indeed, continuous structure analysis revealed a tradeoff between the case dominant Proteobacteria phyla and Bacteroides and Firmicutes, which were only abundant in a subset of cases (Figure S15). A proportion of reads, however, could not be assigned at the Phylum level for both the case (16.4%) and follow-up (13.5%) samples.

**Figure 4:**
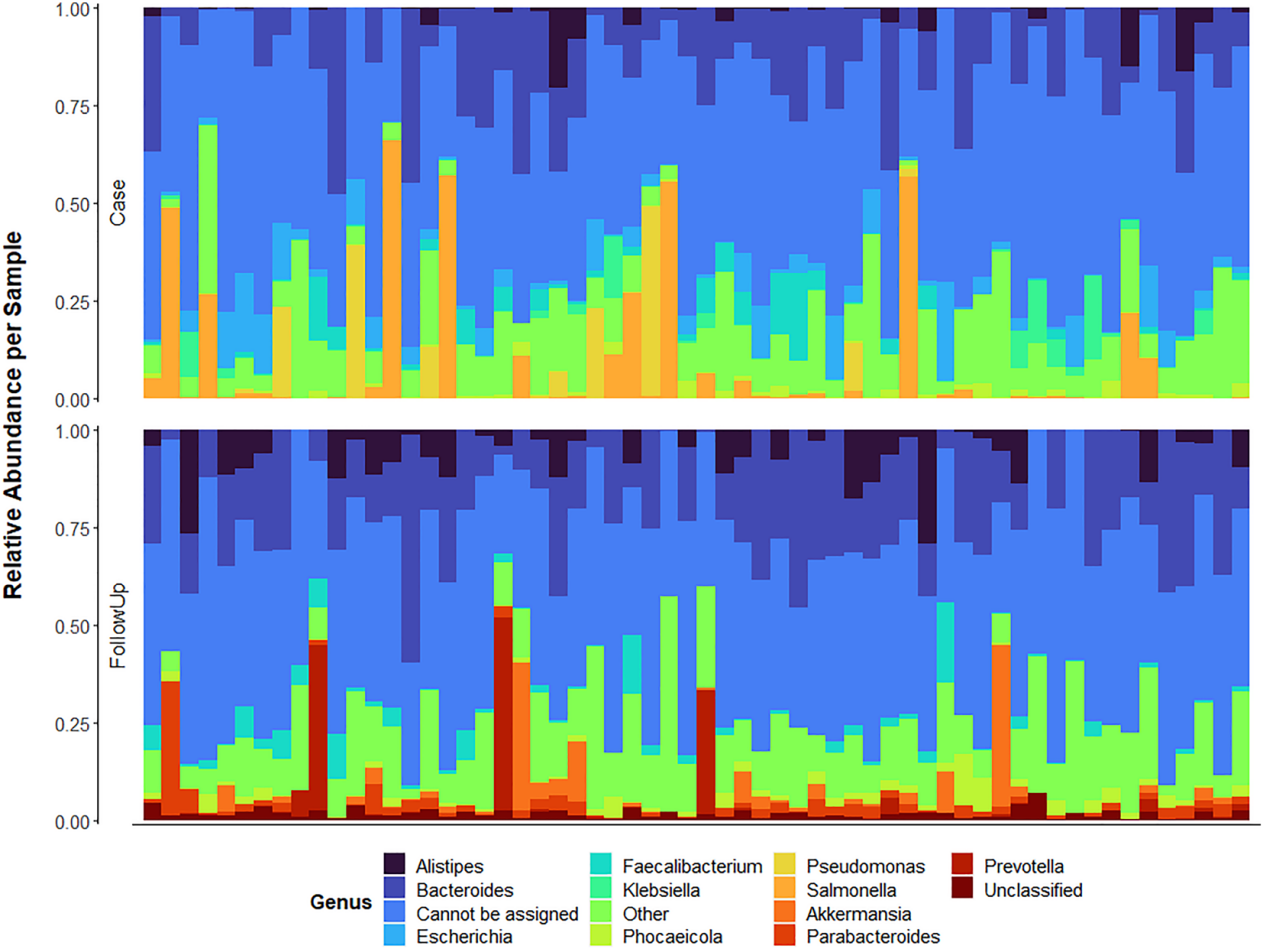
Relative abundance of microbial genera differ between cases and follow-ups. The top-10 microbial genera with the greatest average relative abundance among cases or follow-ups is shown with each column representing the microbiome from one individual. Columns are ordered by their sample pairing, meaning that the column position for each facet of the plot refers to the same individual either during (Case; Top) or after (FollowUp; Bottom) enteric infection. Relative abundances were determined using raw gene abundances that had been normalized by the approximate number of genome equivalents in the sample as determined using MicrobeCensus^17^.

At the genus level, cases and follow-ups both had a high proportion of unclassifiable reads (case = 50.1%; follow-up = 46.9%). Beyond this, *Bacteroides* was, on average, the most prevalent across both cases and follow-ups (14.5% and 18.7%, respectively) (Fig. 4). In cases, patterns of relative abundance differed by infecting pathogen. Among individuals with *Campylobacter* and *Shigella* infections, the genus *Escherichia* comprised a high relative abundance (5.0% and 10.6%, respectively). *Salmonella* displayed the greatest proportion of reads (14.5%) in cases with *Salmonella* infection (n = 29), followed by *Bacteroides* (13.3%) and *Escherichia* (4.5%). Among the three individuals with STEC infections, *Bacteroides* (9.3%) and *Roseburia* (5.8%) species comprised the greatest proportion of genera, with *Escherichia* totaling just 1.9% of all reads. Across all cases, it was evident that members of the *Enterobacteriaceae* family had elevated relative abundance, which was confirmed in the differential abundance (Figure S17) and continuous structure analyses (Figure S15). Specifically, *Escherichia* (coef = − 0.156; q-value = 0.0021) was a predominant genus among cases regardless of infectious agent and was mainly represented by *Escherichia coli* (coef = -0.146; q-value = 0.0082). The *Shigella* genus (coef = -0.057; q-value = 0.0059) comprising three species (*S. sonnei, S. flexneri,* and *S. dysenteriae*) was also overrepresented across cases (Figure S18) as was *Enterobacter* (coef = − 0.020; q-value = 1.10e−08) and *Citrobacter* (coef = − 0.017; q-value = 8.07e−06). Interestingly, *Campylobacter* was not notably abundant among the 24 individuals with *Campylobacter* infections (0.59%). This lack of detection could be due to the cases rapidly clearing the infection prior to stool collection, which would not be surprising given the transient nature of this pathogen in the gut^31^.

In follow-ups, both the Bacteroidetes and Firmicutes populations rebounded and predominated during recovery (49.3% and 26.9%, respectively), as was confirmed in the differential abundance analysis (Bacteroidetes (coef = 0.305; q-value = 1.87e−05); Firmicutes (coef = 0.199; q-value = 4.61e−07)). Across follow-ups, notable signatures of Bacteroidetes genera such as *Bacteroides* (18.7%), *Alistipes* (5.0%) and *Prevotella* (2.5%) were observed as well as the genus *Akkermansia* (2.8%) of Verrucomicrobia, and the Firmicutes genus *Faecalibacterium* (2.4%). These taxonomic patterns among follow-ups closely mirror the microbiota composition of healthy controls assessed in our prior study^7^. Similarly, the relative abundance of these genera varied slightly depending on which infection follow-ups had recovered from. Both *Bacteroides* and *Alistipes*, however, were consistently in the top-three most proportionally abundant genera among follow-ups regardless of the infectious agent. In addition, *Akkermansia* was more abundant among individuals who had recovered from STEC infections (15.7%) relative to those who were infected with *Campylobacter* (0.74%), *Salmonella* (3.3%), or *Shigella* (1.7%). Importantly, relative abundances of genera corresponding to the infecting pathogens were nearly negligible in follow-ups.

### Different ARG-harboring microbial hosts were detected in case and follow-up samples

**Figure 5:**
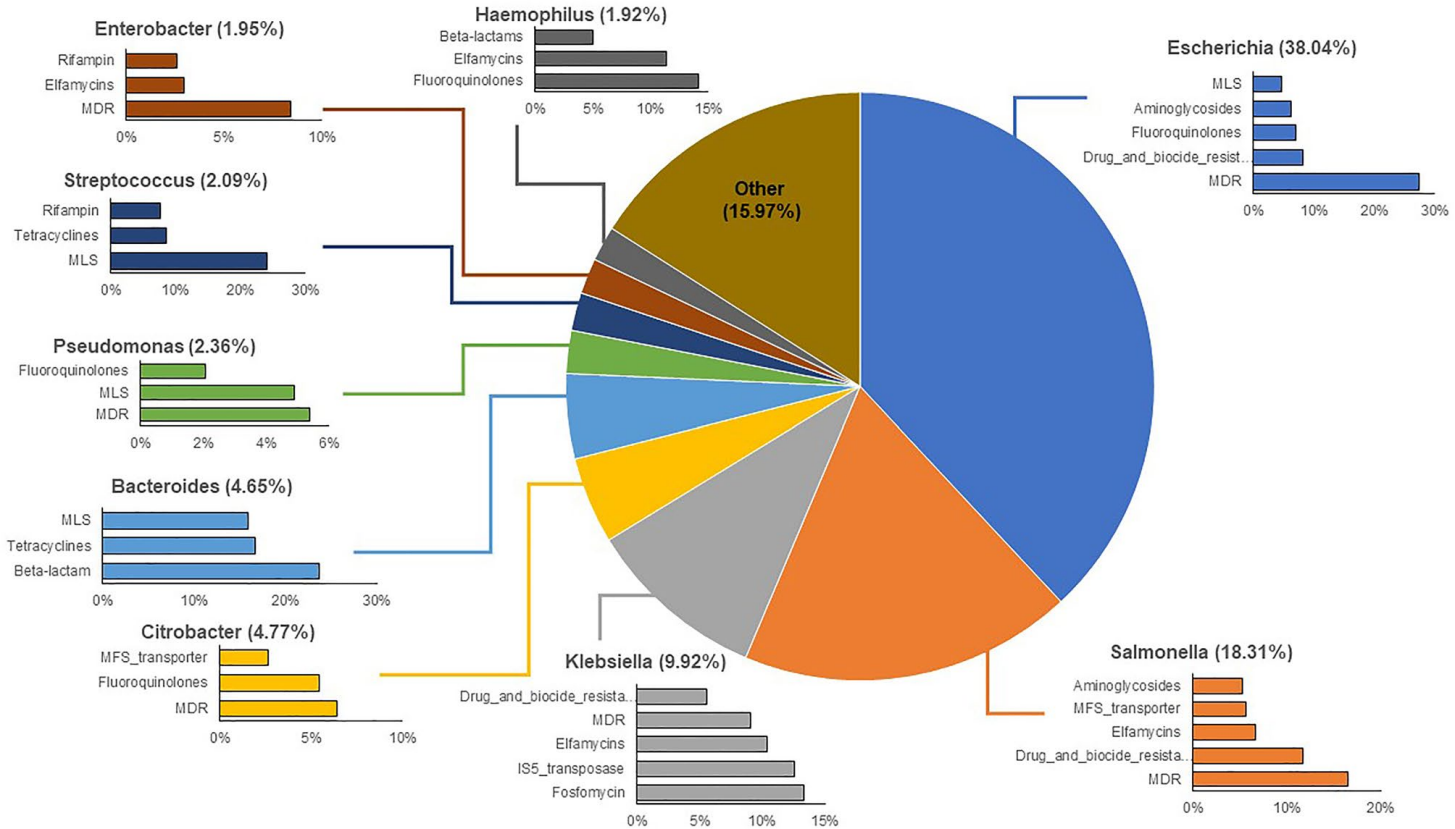
The top-10 genera assigned to antibiotic resistance gene (ARG)-carrying contigs (ACCs) in case samples. The percentages associated with each genus indicate the percent of ACCs assigned to that genus. Each bar chart associated with a genus displays the top-5 or top-3 ARG classes affiliated with that particular genus on the ACCs.

In cases, ACCs, on average, were primarily attributed to *Escherichia* (38.0%) followed by *Salmonella* (18.3%) and *Klebsiella* (9.9%) (Fig. 5). Of the *Escherichia*-associated ARGs, 27.4% were assigned to MDR on average, though ARGs relevant to drug and biocide resistance (8.1%), fluoroquinolone resistance (7.1%), and aminoglycoside resistance (6.2%) were also identified. Comparatively, the *Salmonella*-associated ACCs mostly contained genes for MDR and drug and biocide resistance (16.5% and 11.7%, respectively), while the *Klebsiella* ACCs harbored an array of fosfomycin resistance genes (13.3%) followed by transposase genes in the IS5 family (12.6%). *Klebsiella* ACCs also contained ARGs for elfamycin resistance (10.4%) and MDR (9.08%).

**Figure 6:**
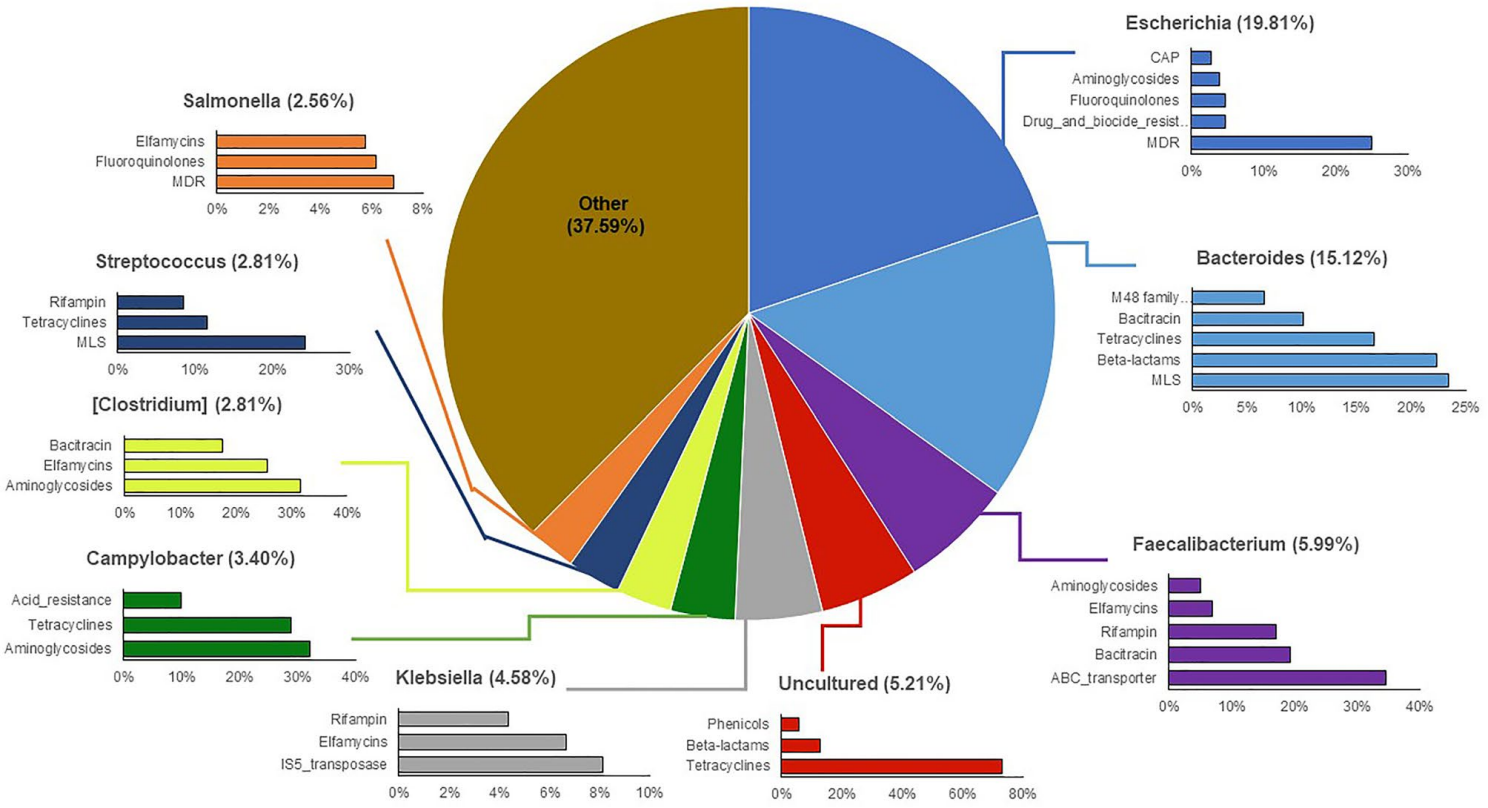
The top-10 genera assigned to antibiotic resistance gene (ARG)-carrying contigs (ACCs) in follow-up samples. The percentages associated with each genus indicate the percent of ACCs assigned to that genus. Each bar chart associated with a genus displays the top-5 or top-3 ARG classes affiliated with that particular genus on the ACCs.

Although the most prominent genus in follow-up ACCs was also *Escherichia* (19.8%), the next most prevalent genera were classified as *Bacteroides* (15.1%) and *Faecalibacterium* (6.0%) (Fig. 6). Notably, the array of ARGs harbored in the *Escherichia*-associated ACCs was nearly identical to cases, with MDR genes predominating (25.1%) followed by resistance to drugs and biocides (4.7%), fluoroquinolones (4.7%), and aminoglycosides (3.8%). Of the *Bacteroidetes*-associated ACCs, genes for MLS, beta-lactam, and tetracycline resistance were the most common. The 5.2% of the ACCs that could not be classified and represented an “Uncultured” taxon harbored ARGs for tetracyclines, beta-lactams and phenicols.

### Microbes linked to case infections harbor ARGs during and after recovery

Differences in ACCs were also identified after stratifying by the bacterium linked to each infection. Among the 23 cases with *Campylobacter* (n = 23) infections, for instance, the genera comprising the greatest proportion of ACCs were *Escherichia* (42.8%), *Klebsiella* (10.0%), and *Salmonella* (7.1%). Upon recovery, however, cases with *Campylobacter* infections most often had ACCs representing *Bacteroides* (18.3%), followed by *Escherichia* (17.3%) and *Faecalibacterium* (6.8%). It is also notable that *Campylobacter* was in the top-20 genera represented on ACCs, comprising 1.2% and 3.8% of all genera assigned to ACCs in cases and follow-ups, respectively. Nonetheless, *Campylobacter-*associated ARGs in case samples conferred resistance to tetracyclines (27.6%), aminoglycosides (9.9%), and rifampin (8.3%), whereas the *Campylobacter* ACCs in follow-ups conferred resistance to tetracycline (29.0%), aminoglycoside (27.0%) and MLS (10.3%) antibiotics. Genes encoding resistance to aminoglycosides were 2.7 times more prevalent among *Campylobacter* ACCs in the follow-up samples relative to the case samples.

In the 29 cases with *Salmonella* infections, most ACCs were taxonomically assigned to *Escherichia* (32.4%)*, Salmonella* (31.0%), and *Klebsiella* (7.9%) as opposed to the follow-ups in which *Escherichia* (20.7%), *Bacteroides* (14.2%), and *Faecalibacterium* (6.3%) predominated. The most common ARGs detected in *Salmonella* ACCs are important for multi-compound resistance, including drug and biocide resistance (14.1%), and MFS transporters (13.1%), which can have MDR effects or high specificity to certain classes. ARGs for drug, biocide, and metal resistance (7.6%) were also identified. Among the follow-up samples, the most prevalent class within *Salmonella*-associated ACCs was RND efflux transporters (9.3%), followed by MFS transporters (6.8%) and fluoroquinolone resistance genes (6.3%).

### Clinically important ESBLs are present after recovery from enteric infections

In all, 49 distinct genes conferring beta-lactam resistance were identified; these encode class A, C, and D beta-lactamases (Table S2). Moreover, 11 (22.4%) distinct genes encoding ESBL production were detected conferring resistance to multiple beta-lactam antibiotics. Among these ESBL genes, those belonging to the CepA family of class A beta-lactamases were most prevalent, occurring in 19 case and 13 follow-up samples; each gene was taxonomically assigned to *Bacteroides*. ESBL genes of the OXA family, which included OXA-1, OXA-50, OXA-51, and OXA-61, were also detected; however, each gene was attributed to a different microbial host in the ACC analysis and was only found in 2–3 individuals. Although the OXA-61 family of class D beta-lactamases was found in *Campylobacter*, it was only detected in two of the 23 cases with *Campylobacter* infections. *Klebsiella* was associated with OXY genes in two cases as well as the SHV family of class A beta-lactamases in eight case and two follow-up samples. Genes representing the ADC family of class C ESBLs harbored by *Acinetobacter* were also detected.

Among other relevant non-ESBL beta-lactamases, the BlaEC family of class C beta-lactamases was detected in 49 cases and 19 follow-ups and was primarily associated with genus *Escherichia*. Genes encoding the CfxA family of class A broad-spectrum beta-lactamases were also frequently detected in *Bacteroides* and *Prevotella*. Of those associated with *Bacteroides*, CfxA genes were found in 46 cases and 48 follow-ups, while CfxA genes associated with *Prevotella* were found in five cases and nine follow-ups. Genes encoding the broad CMY-family of class C beta-lactamases were also detected and assigned to *Salmonella* in three cases and two follow-ups. Relatedly, the CMY-2 family of class C beta-lactamases was identified within *Citrobacter* and *Salmonella;* eight cases had CMY-2 genes associated with *Citrobacter*, while two cases had CMY-2 genes assigned to *Salmonella.* CMY-2 genes were also linked to *Citrobacter* in three follow-up samples and to *Salmonella* in one. Lastly, genes for the general subclass A2 of class A beta-lactamases were found in *Bacteroides* in both the cases (n = 45) and follow-ups (n = 47), while the general “class A beta-lactamase” gene was detected in nine other genera including *Atlantibacter*, *Bacillus, Burkholderia, Clostridium, Proteus, Salmonella, Yersinia, Escherichia,* and *Klebsiella*.

## Discussion

The human gut microbiome, when disrupted by an infectious pathogen, can drastically change in composition taxonomically, genetically, and functionally^32^. In most instances, pathogen invasion leads to a state of dysbiosis linked to a decrease in gut microbiota diversity^4,33^. Our study supports these findings, as markedly lower microbiota diversity was observed among cases during infection than after recovery regardless of the bacterial pathogen causing infection. The observed shifts in microbiota composition post-recovery are indicative of gut health, as healthy family members (controls) and follow-ups had more similar taxonomic profiles than the cases. In addition to the increased microbiota diversity post-recovery, specific taxonomic signatures such as enhanced abundance of Bacteroidetes and Firmicutes, were observed. For instance, members of *Bacteroides, Prevotella,* and *Phocaeicola* as well as *Faecalibacterium, Roseburia,* and *Ruminococcus* were found, which have been shown to play influential roles in maintaining gut homeostasis and metabolic health^34–36^. By contrast, the cases were defined primarily by members of Proteobacteria such as *Escherichia, Salmonella, Shigella,* and *Klebsiella*, which have been linked to acute enteric disturbances as well as prolonged dysbiosis and long-term disease outcomes^37^. Collectively, the taxonomic data also provide support for common microbiota disturbances regardless of the pathogen causing the infection, as the differing abundances were similar to those described in our prior study of patients with *Campylobacter* infections^7^.

The opposite was true for the collection of ARGs, as cases had greater resistome diversity during infection than after recovery. Because shifts in microbial composition inherently influence the presence and abundance of ARGs harbored by microbes within a community, this finding is not surprising. Among the key differences observed, cases had more multi-compound and multi-drug resistance genes during infection than post-recovery, whereas tetracycline, MLS, and aminoglycoside resistance genes were more abundant in the recovered (follow-up) sample. Diverse sets of ARGs have previously been found in otherwise healthy individuals as well^7,38,39^, providing additional support for the human gut as an important reservoir of antibiotic resistance determinants^11^. Although a subset of patients (n = 2) and follow-ups (n = 5) had taken antibiotics, which are known disruptors of microbial communities, the sample size was too small to determine whether specific antibiotics impacted the ARG or taxonomic profiles. Nonetheless, the microbiota PCoA showed that antibiotic use significantly influenced the distribution of points in the ordination for microbiota composition but not ARG abundance. Additional studies are therefore needed to understand how specific antibiotics impact the microbiota composition before and after infection. The same was true for age in years, which influenced the distribution of points in the microbiota and ARG PCoA. Because age has been shown to influence the composition of the microbiota^40,41^, future studies using larger sample sizes are required in order to stratify the profiles by age group.

Intriguingly, the PCoA point distribution for microbiota abundances was also influenced by the number of days between samplings, or the follow-up period. Upon further inspection, a subset of five follow-up samples were more closely related to the case microbiota and resistome samples in the PCoA. Because these patients had an average number of 110 days since infection, which did not differ from the overall mean (n = 108 days), other factors likely contributed to the case-like taxonomic profiles observed. Indeed, four patients were either < 10 or > 50 years of age and two of these individuals were hospitalized. Since children and older individuals typically have an enhanced risk of developing more severe disease^42,43^, these patients could have experienced lengthier infections than other members of the sample cohort. The same is true for those who were hospitalized and hence, the microbiota may have not fully recovered at the time of follow-up sampling. The complete level of microbiome recovery, however, could not be deduced for any of the patients since we did not evaluate the gut microbiome in the same patients prior to infection. It is likely that the state of the microbiome prior to infection as well as its resilience to disturbances will vary across individuals and greatly impact the trajectory of disease and recovery. Implementation of a more rigorous longitudinal study is therefore needed.

In the host-tracking analysis, we demonstrated that specific microbial taxa were more likely to harbor ARGs during infection. *Escherichia*, for instance, was a prominent host in the cases regardless of the pathogen linked to the infection. Specifically, *Escherichia* comprised an average of 38% of all ACCs, with most genes being important for MDR or multi-compound resistance. This result is not surprising given the increased abundance of *Escherichia* observed during infection. Expansion of *Escherichia* and Enterobacteriaceae in general, was previously suggested to be linked to inflammation in the gut^44^, which was also shown to augment HGT rates between commensal and pathogenic members of this family^45^. Moreover, as the level of MDR increases within a population, so too does the number of integrons, which were also shown to persist among commensal *E. coli*^46^. This enhanced mobility and maintenance of resistance determinants are key contributors to the emergence of resistant pathobionts^3,47^.

Evidence of ARGs harbored by genera linked to the acute infections was also observed, indicating that some pathogens bring resistance genes into the gut during infection. In patients with *Salmonella* infections, for instance, *Salmonella* accounted for ~ 31% of all ACCs compared to the overall case average of 18%, with most genes encoding MDR or drug and biocide resistance. Co-selection for resistance to antibiotics, metals, and biocides has been previously documented in *Salmonella* and other foodborne pathogens^48^. This evidence is supported by data generated in a co-occurrence network analysis despite being a less robust approach^49^. Notably, a *Salmonella*-specific subnetwork comprised of various metal, biocide, and MDR genes was identified among *Salmonella* cases (Figure S19). These findings indicate that the different *Salmonella* pathogens brought similar ARGs into the microbial communities at the time of infection. This subnetwork was not detected in the co-occurrence network generated for the *Campylobacter* cases alone despite the identification of ACCs attributes to *Salmonella* (Figure S20). The detection of *Salmonella* in the *Campylobacter* cases is interesting but not unprecedented, as previous studies have identified polymicrobial infections involving multiple enteric pathogens^50^ including *C. jejuni* and *Salmonella*^51^. It is therefore possible that only the *Campylobacter* was recovered from these cases at the time of sampling. Future studies that apply whole-genome sequencing to the bacterial pathogens recovered from each sample are needed to determine the diversity and frequency of those ARGs that were introduced into each gut community by the infecting pathogen.

In the follow-up samples, *Escherichia* still accounted for the greatest proportion (~ 20%) of all ARG-carrying contigs, which mostly contained MDR genes; however, the proportion was 1.9 times less than that observed during infection. Unlike the cases, *Bacteroides* was the second most important genus accounting for ~ 15% of the ARG-carrying contigs at recovery with MLS, beta-lactam, and tetracycline resistance genes predominating. Members of Bacteroidetes and Firmicutes have previously been linked to high levels of tetracycline and erythromycin resistance carrying genes such as *tetQ* as well as *ermF* and *ermG*, respectively^52^. These genes were previously suggested to be maintained in microbial host populations even in the absence of antibiotic selection, thereby enhancing the likelihood of HGT^52^. Although resistance to beta-lactam antibiotics has been documented, variation in resistance rates has been observed across species and geographic locations, particularly for the beta-lactamase producers^53,54^.

Indeed, the transfer and acquisition of genes encoding beta-lactamase production is of great concern. During enteric infection, we detected 11 distinct ESBLs that varied in frequency among the cases, although this number may underestimate the actual diversity as not all sequences could be assigned a class designation^55^. Our finding that *Klebsiella* and *Escherichia* both harbored ESBLs in case and/or follow-up samples calls attention to the documented capacity of these genera to transfer genes across species or clonal lineages^56^. Importantly, ESBL-producing *Escherichia coli* have been documented in healthy children and adults^57^ and beta-lactamase genes are increasingly prevalent in the human gut, even among healthy subjects^58^. In our study, *Klebsiella* was a prominent ARG carrier in 9.2% and 4.6% of ACCs in the cases and follow-ups, respectively, and was associated with a high occurrence of the IS5 family of transposases. The identification of a genomic element with capacity to transfer ARGs is notable, particularly to other members of *Enterobacteriaceae* that have contributed to the widespread distribution of ESBL genes^2,59^. Although relevant to the spread of ESBLs and other ARGs, HGT could not be confirmed in our study based on the detection of a gene in two genera at different time points. Hence, future work should employ more rigorous methods such as enhanced sequencing depth and characterizing sequence-level similarity among ARGs, to more confidently infer gene transfer between and within genera.

Other limitations related to the ACC analysis include the potential for misclassifying ARGs found on plasmids even though they were previously shown to contain taxonomic information regarding the host microbe^60^. Because assembly of short-read sequences can inaccurately characterize plasmids and other MGEs^61^, deeper sequencing is needed to generate more complete assemblies and avoid misclassifying microbial hosts. Use of bioinformatic tools that focus on plasmid sequence analysis would also be informative if deeper sequencing methods are applied. Moreover, multiple ARGs were attributed to “uncultured” microbes. This finding, in addition to the large proportion of unassigned reads, also highlight the need for more comprehensive databases that can accurately predict microbial host taxonomies. Relatedly, our use of an amino acid-based taxonomic classifier for short reads (Kaiju) may have resulted in the overestimation of known taxa or genes, a limitation that may falsely inflate our measure of species richness and diversity^62^. In addition, the greatest proportion of unassigned reads was observed for lower taxonomic ranks (e.g., Genus, Species), suggesting that nucleic acid- or marker gene-based classification tools such as Kraken 2^63^ or MetaPhlAn 4^64^, respectively, may be better for taxonomic annotation. Since all our samples were processed with Kaiju at the same resolution, however, any loss of taxonomic information should be consistent across samples. Nonetheless, these alternative annotation strategies, used in conjunction with deeper sequencing methods, may be needed to further characterize gut communities. Because the ACC analysis relies on classifying microbial hosts based on co-occurrence of an ARG and its taxa on the same contig, alternative methods such as Single-molecule Real-time sequencing, could also be applied to address this issue in future studies. Finally, this study was performed before the inclusion of sequencing controls was common, and therefore, positive and negative controls were not included. These controls are required to appropriately remove potential contaminating sequences, which was not performed here. This omission could alter certain interpretations, particularly for the less abundant taxa or genes and may explain some of the batch effects observed by run.

Despite these limitations, this study provides important data about alterations in the gut microbiota and resistome among patients with acute enteric infections caused by four bacterial pathogens. Our work also captures the relative restoration of a “healthy” gut following recovery from these infections in that the communities rebound to be more similar to the household (uninfected) controls. Such findings are needed to guide the development of targeted intervention strategies and therapeutic options aimed at rehabilitating a dysbiotic gut. Future work should focus on understanding the trajectory of recovery as it pertains to the presence and dissemination of drug resistance. Importantly, characterizing the interactions between microbial hosts, ARGs, and MGEs during the process of recovery is crucial to our understanding of how enteric infection impacts such dissemination.

### Supplementary Information

Supplementary Information.

## Data Availability

Sequencing reads were deposited in the National Center for Biotechnology Information (NCBI) sequence read archive (SRA) database under BioProjects PRJNA862908 and PRJNA660443 (BioSamples SAMN29999523 to SAMN29999673 and SAMN15958881 to SAMN15958950, respectively). Bioinformatic scripts were described previously^7^ and are available at: https://github.com/ZoeHansen/PAPER_Hansen_ScientificReports_2023.
