# Supplementary material for: Recovery of the gut microbiome following enteric infection and persistence of antimicrobial resistance genes in specific microbial hosts
Source: Sci Rep. 2023 Sep 19;13:15524. doi: 10.1038/s41598-023-42822-7 (PMC10509190; doi:10.1038/s41598-023-42822-7)
Supplement: Supplementary file 1 — Supplementary Information. [file 41598_2023_42822_MOESM1_ESM.pdf]

Supplementary Information

Recovery of the gut microbiome following enteric infection and persistence of antimicrobial resistance genes in specific microbial hosts

Zoe A. Hansen,[1] Karla Vasco,[1] James T. Rudrik,[4] Kim T. Scribner,[2]

Lixin Zhang,[3] Shannon D. Manning[1*]

Michigan State University, Departments of [1]Microbiology and Molecular Genetics, [2]Fisheries and Wildlife, and [3]Epidemiology and Biostatistics, E. Lansing, MI 48824; and [4]the Michigan Department of Health and Human Services, Bureau of Laboratories, Lansing, MI 48906

E-mail addresses:

hansenzo@msu.edu; vascokar@msu.edu; microbehunter@yahoo.com; scribne3@msu.edu; lxzhang@msu.edu; mannin71@msu.edu

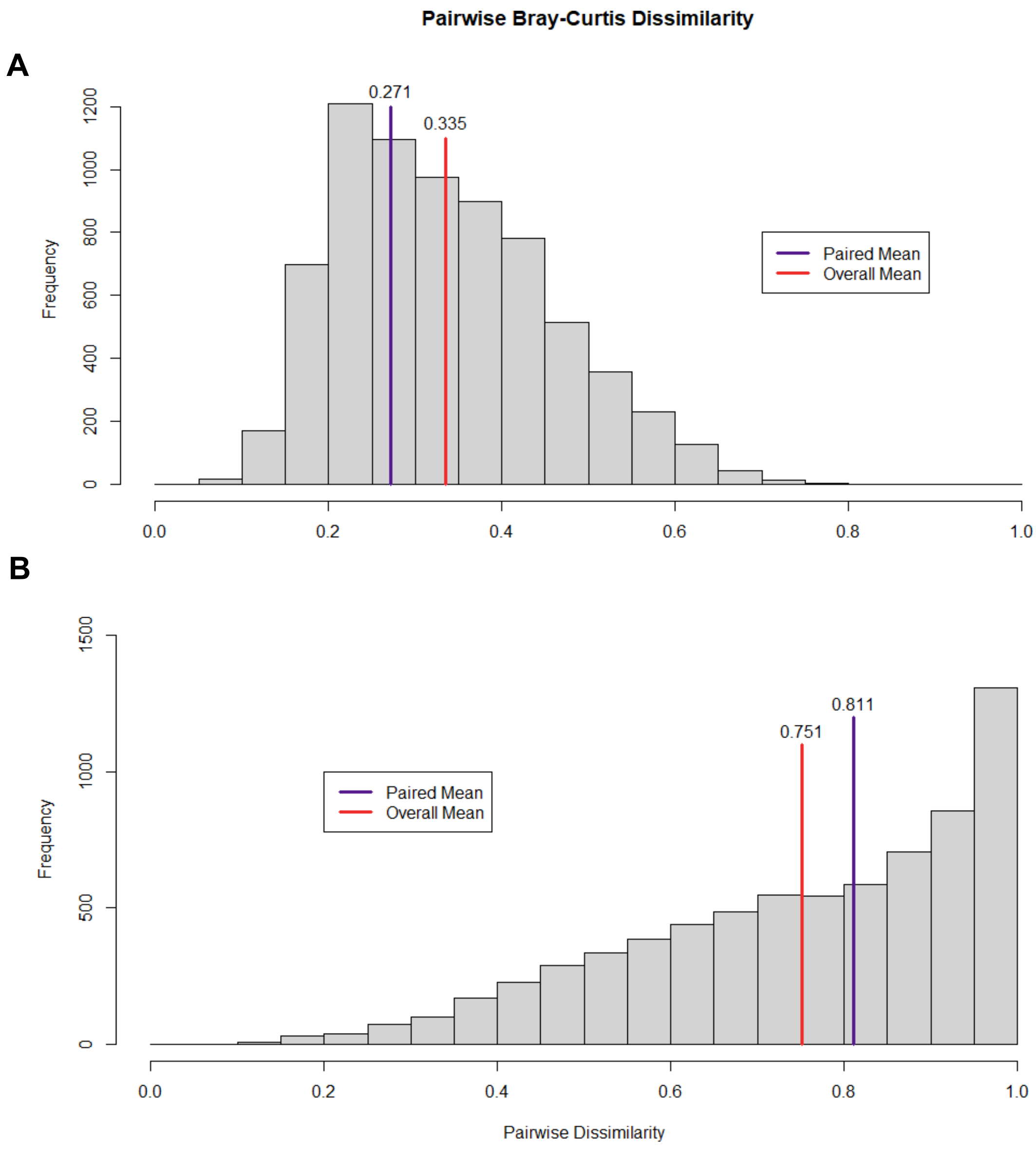

Figure S1. Pairwise comparison of Bray-Curtis dissimilarity among cases and follow-ups for the A) the microbiota (species-level) and B) resistome (gene-level) data. Upon construction of the dissimilarity matrix, pairwise dissimilarity scores were output into two separate data frames: one contained all pairwise comparisons and one contained only pairwise comparisons among relevant paired samples. In each plot, the overall mean dissimilarity across all samples is displayed as a red line with the value of the mean oriented above the line (microbiome = 0.335; resistome = 0.751). The mean among paired samples only is shown as a purple line (microbiome = 0.271; resistome = 0.811). A Welch's t-test indicated that the dissimilarity among paired samples was significantly lower than the overall mean for microbiome composition (p = 2.58e-05; two-sided) and was significantly higher than the overall mean for the resistome (p = 0.013; two-sided).

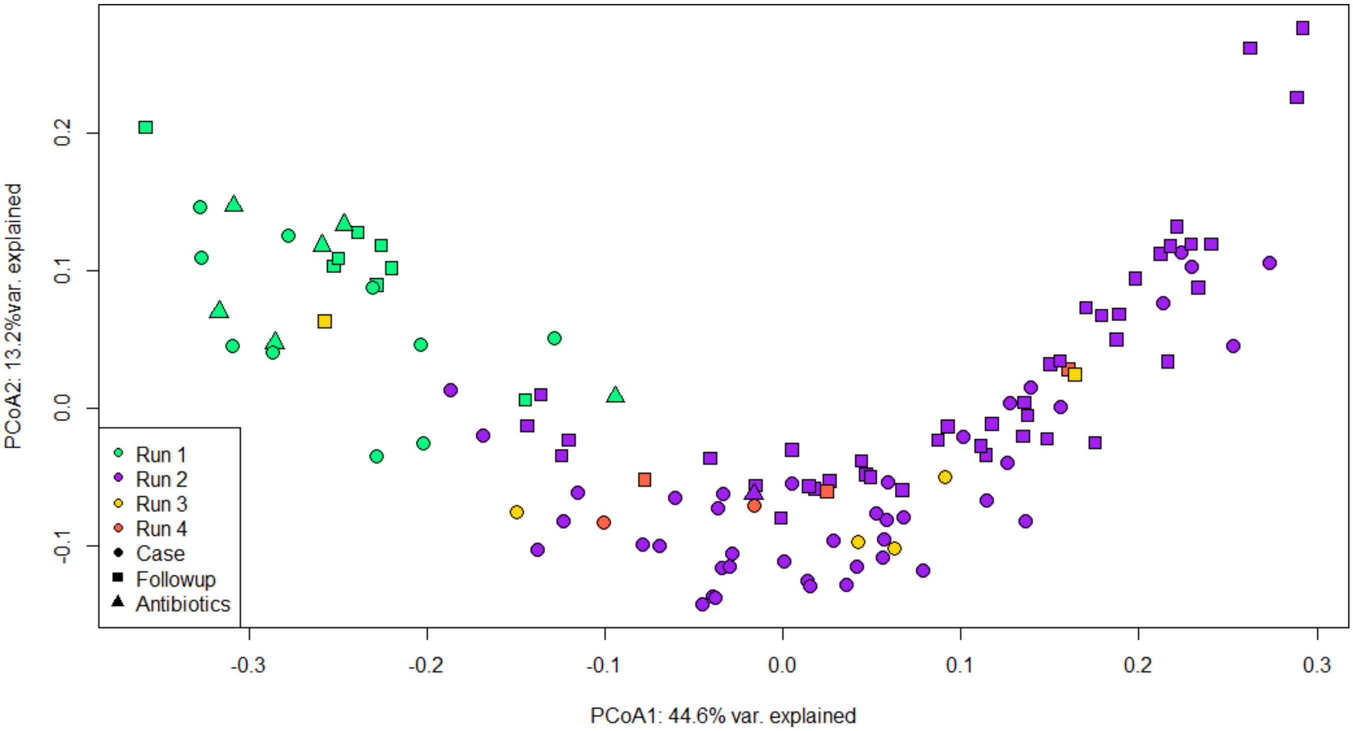

Figure S2. Exploring potential batch effects related to sequencing run using Principal Coordinate Analysis (PCoA) of cases and follow-ups. The plot shows the distribution of cases (circles) and follow-ups (squares) based on Bray-Curtis dissimilarity calculated from species-level abundances of the gut microbiota. The first and second coordinate display the percentage of similarity explained. Points are colored by their corresponding sequencing run: Run 1 (green), Run 2 (purple), Run 3 (yellow), and Run 4 (orange). Samples sequenced in Run 1 cluster separately from those in Runs 2, 3, and 4 along the first axis. Patients that self-reported use of antibiotics two weeks prior to sample collection are indicated by triangles.

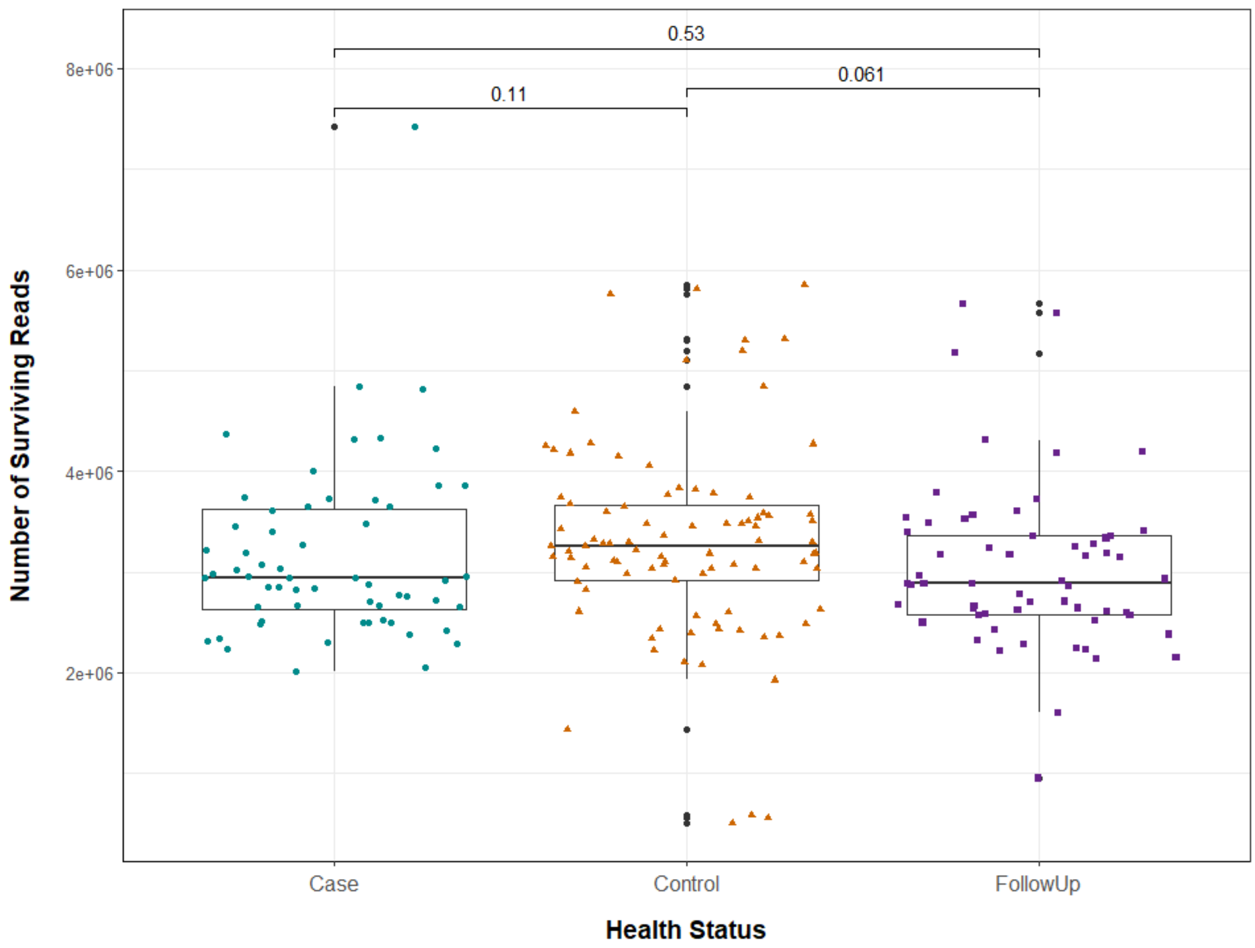

Figure S3. Number of paired-end reads that survive trimming do not differ between sample types. The number of surviving paired-end reads following Trimmomatic are stratified by health status. Case samples are represented by green circles, post-recovery samples ("FollowUp") are shown as purple squares, and household controls ("Control") are shown as orange triangles. Points are offset from the vertical to allow interpretation of all samples. The median of each measure is shown as a thick bar within the box and the first and third quartiles are represented by the bottom and top of the box, respectively. Black circles (in-line with interquartile range lines (vertical) indicate presumed outliers defined as ≥1.5 x IQR; outlier points relate to datapoints already displayed in the plot. P-values were calculated using the Wilcoxon signed-rank test for paired samples and are shown above the comparison bar within each plot.

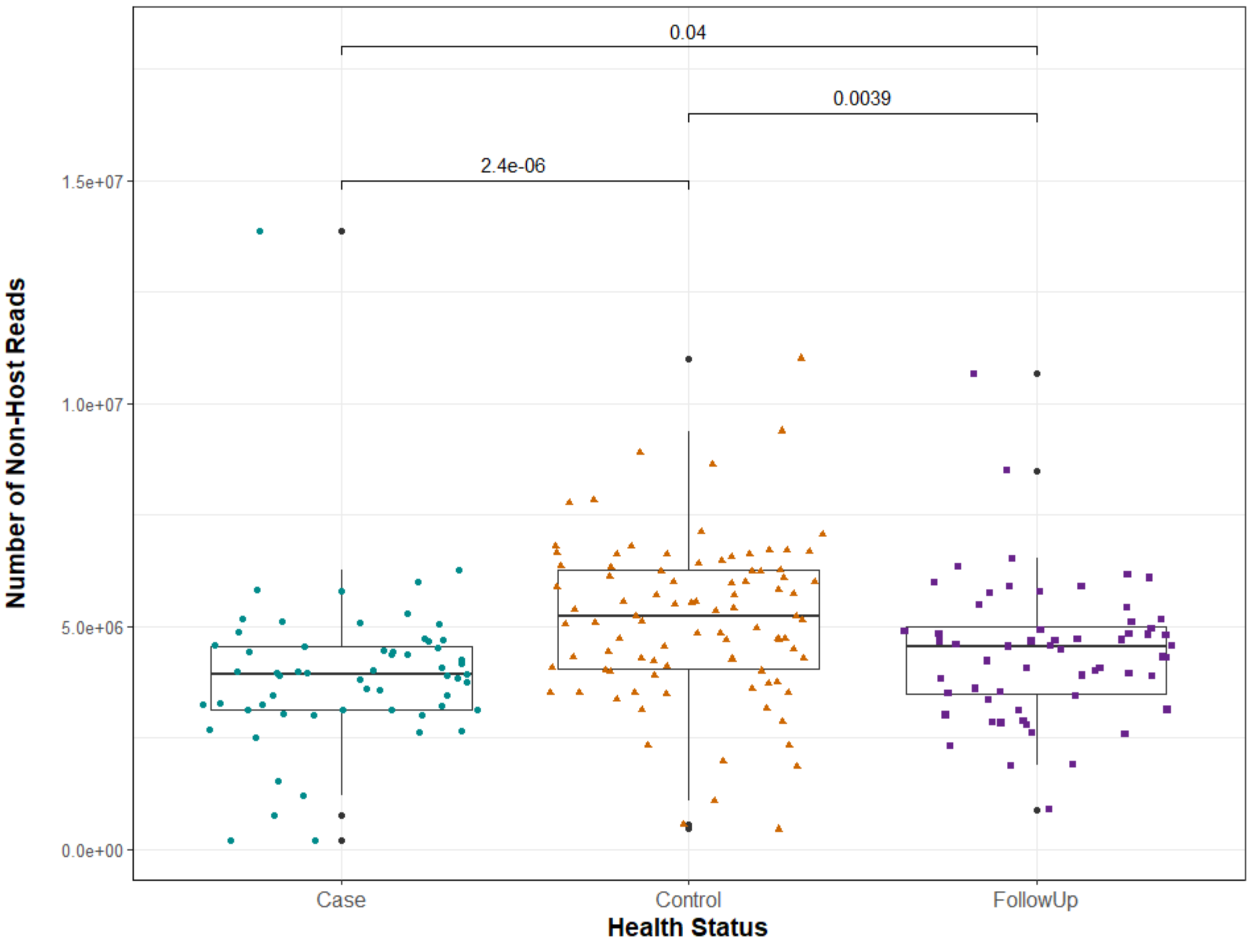

Figure S4. Number of non-host reads significantly differ between sample types. The number of sequencing reads that did not map to the human genome ("Non-Host") are stratified by health status. Case samples are represented by green circles, post-recovery samples ("FollowUp") are shown as purple squares, and household controls ("Control") are shown as orange triangles. Points are offset from the vertical to allow interpretation of all samples. The median of each measure is shown as a thick bar within the box and the first and third quartiles are represented by the bottom and top of the box, respectively. Black circles (in-line with interquartile range lines (vertical) indicate presumed outliers defined as ≥1.5 x IQR; outlier points relate to datapoints already displayed in the plot. P-values were calculated using the Wilcoxon signed-rank test for paired samples and are shown above the comparison bar within each plot.

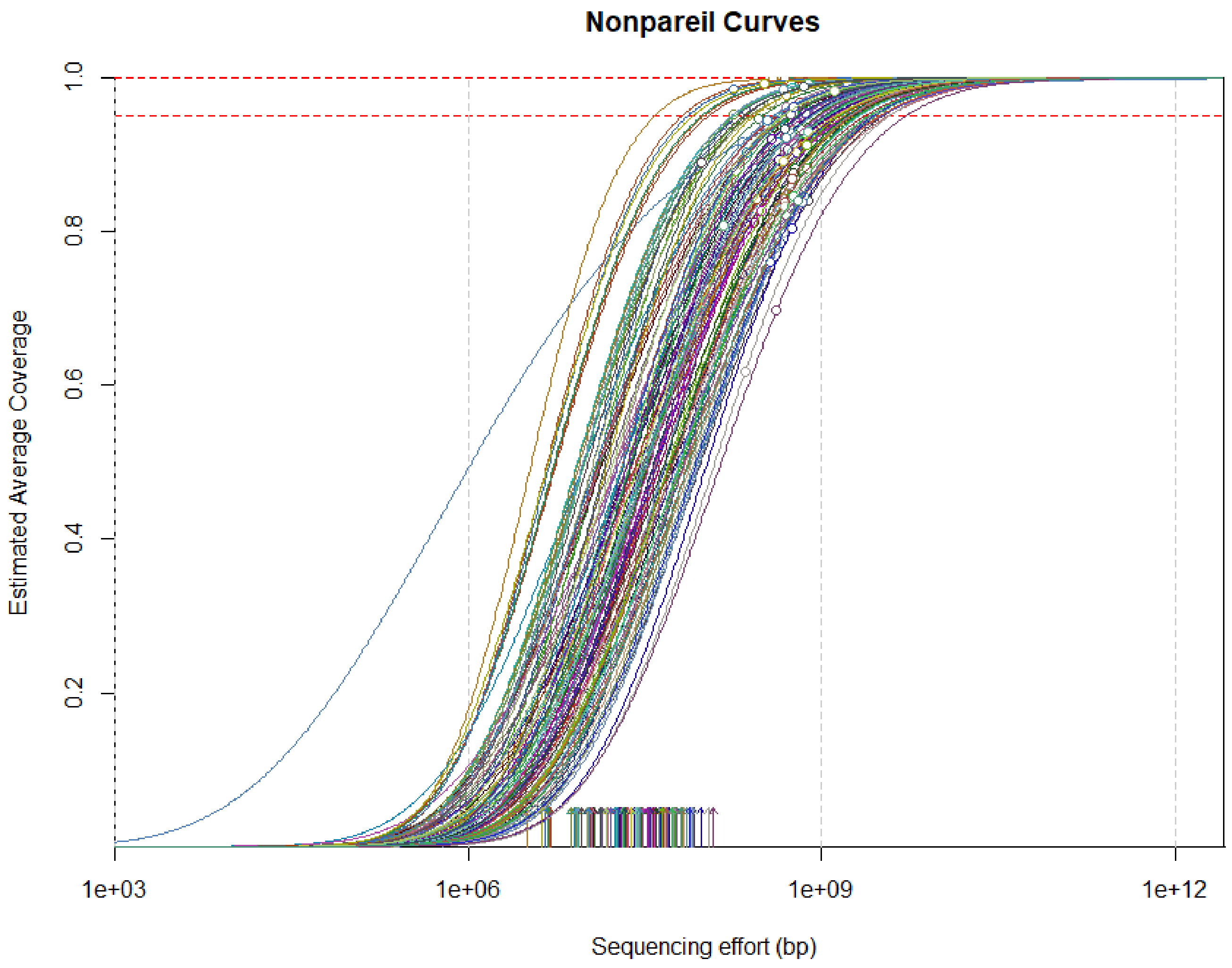

Figure S5. Metagenomic sequencing coverage of short paired-end reads was determined using Nonpareil. The estimated sequencing coverage at increasing sequencing effort (s-curves) is shown as well as the actual coverage of each sample (open circles) for the case (n=60) and follow-up (n=60) samples. Each s-curve represents a single sample; arrows aligned on the x-axis represent the Nonpareil index of sequence diversity, a metric capturing the complexity of microbial communities in sequencing space. The box bordered by dotted red lines encapsulated coverage ranging from 95-100%. The overall mean coverage among cases and follow-ups was 86.3%, with a Nonpareil diversity score of 17.0. Additionally, the estimated sequencing effort among all cases and follow-ups was 4.77e+08 base pairs.

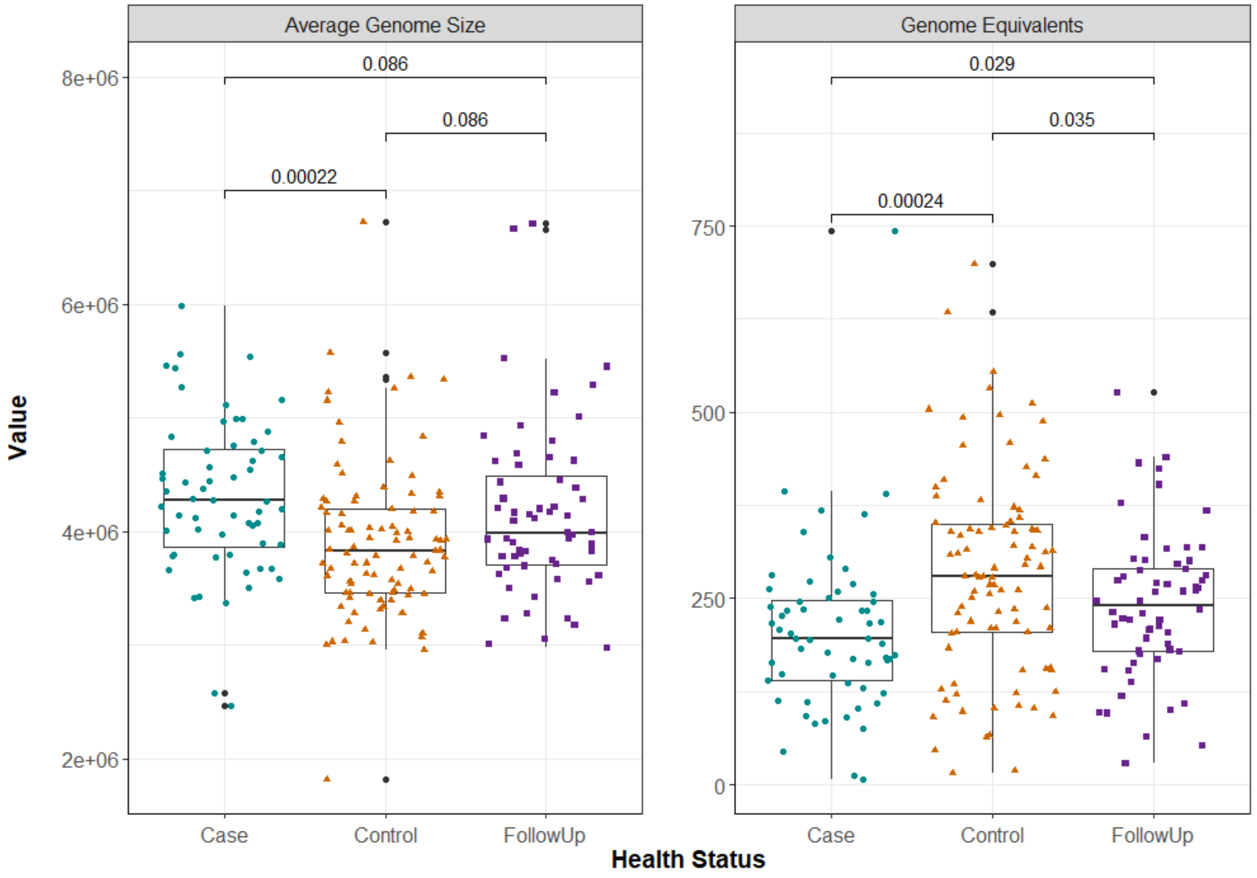

Figure S6. Average genome size (AGS) and estimated number of genome equivalents (GE) for all samples. Each metric is displayed and stratified by health status. Case samples are represented by green circles, follow-up samples are shown as purple squares, and control samples are indicated by orange triangles. Points are offset from the vertical to allow interpretation of all samples. The median of each measure is shown as a thick bar within the box and the first and third quartiles are represented by the bottom and top of the box, respectively. Black circles (in-line with interquartile range lines (vertical) indicate presumed outliers defined as ≥1.5 x IQR; outlier points relate to datapoints already displayed in the plot. P-values were calculated using the Wilcoxon signed-rank test for paired samples and are shown above the comparison bar within each plot.

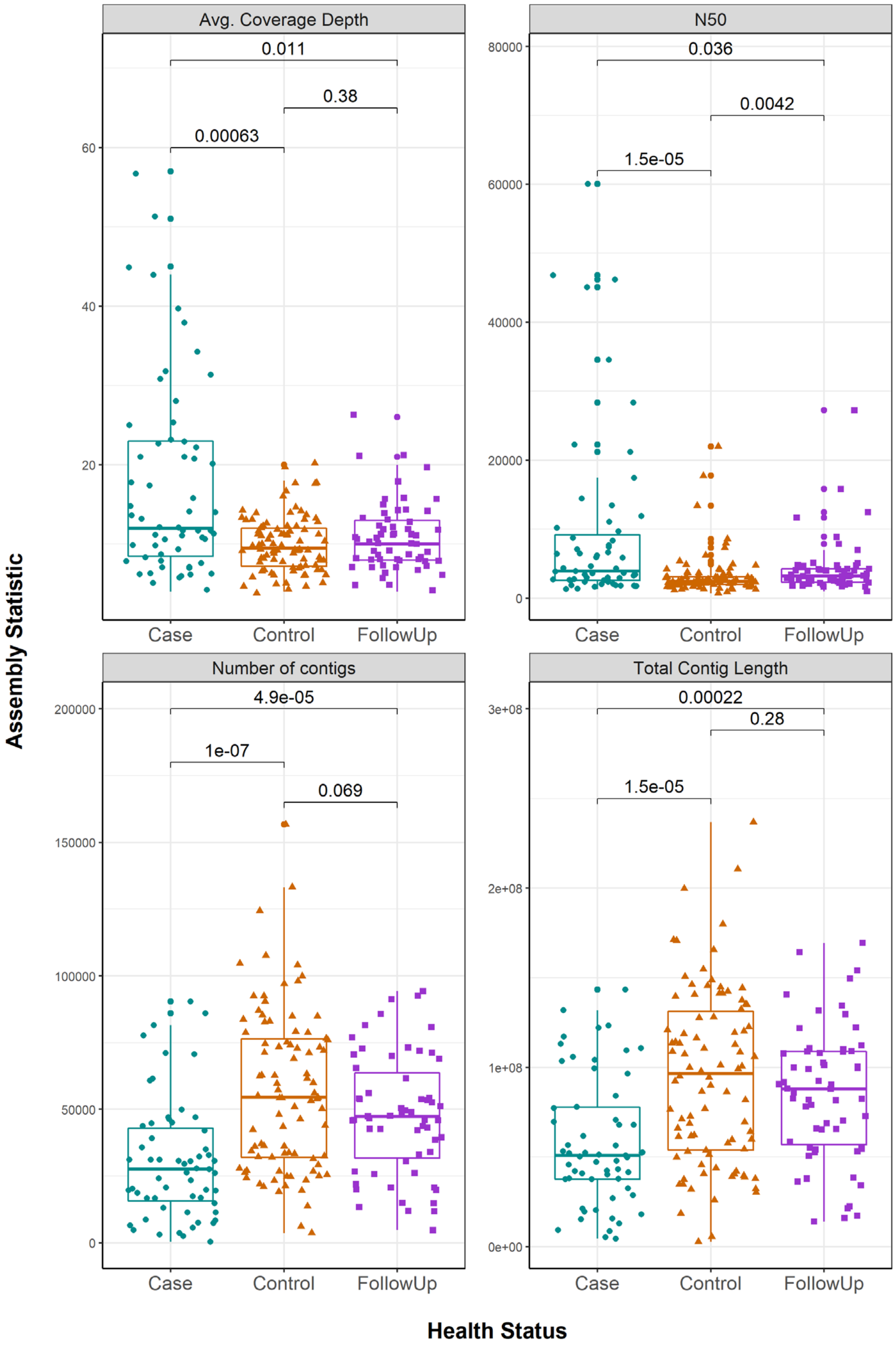

Figure S7. Assembly coverage statistics quantified by the Quality Assessment Tool for Genome Assemblies (QUAST). Four different assembly statistics are shown stratified by health status, with samples represented by green circles (cases), purple squares (follow-ups), or orange triangles (controls). Clockwise from the top-left, the four metrics include: average (Avg.) depth of coverage, the N50 value, number of contigs, and total contig length. In each plot, points are offset from the vertical for clarity. The median is shown as a thick bar within the box (green for cases; purple for follow-ups; orange for controls) and the first and third quartiles are represented by the bottom and top of the box, respectively. P-values were calculated using the Wilcoxon signed-rank test for paired samples and are shown above the comparison bar within each plot.

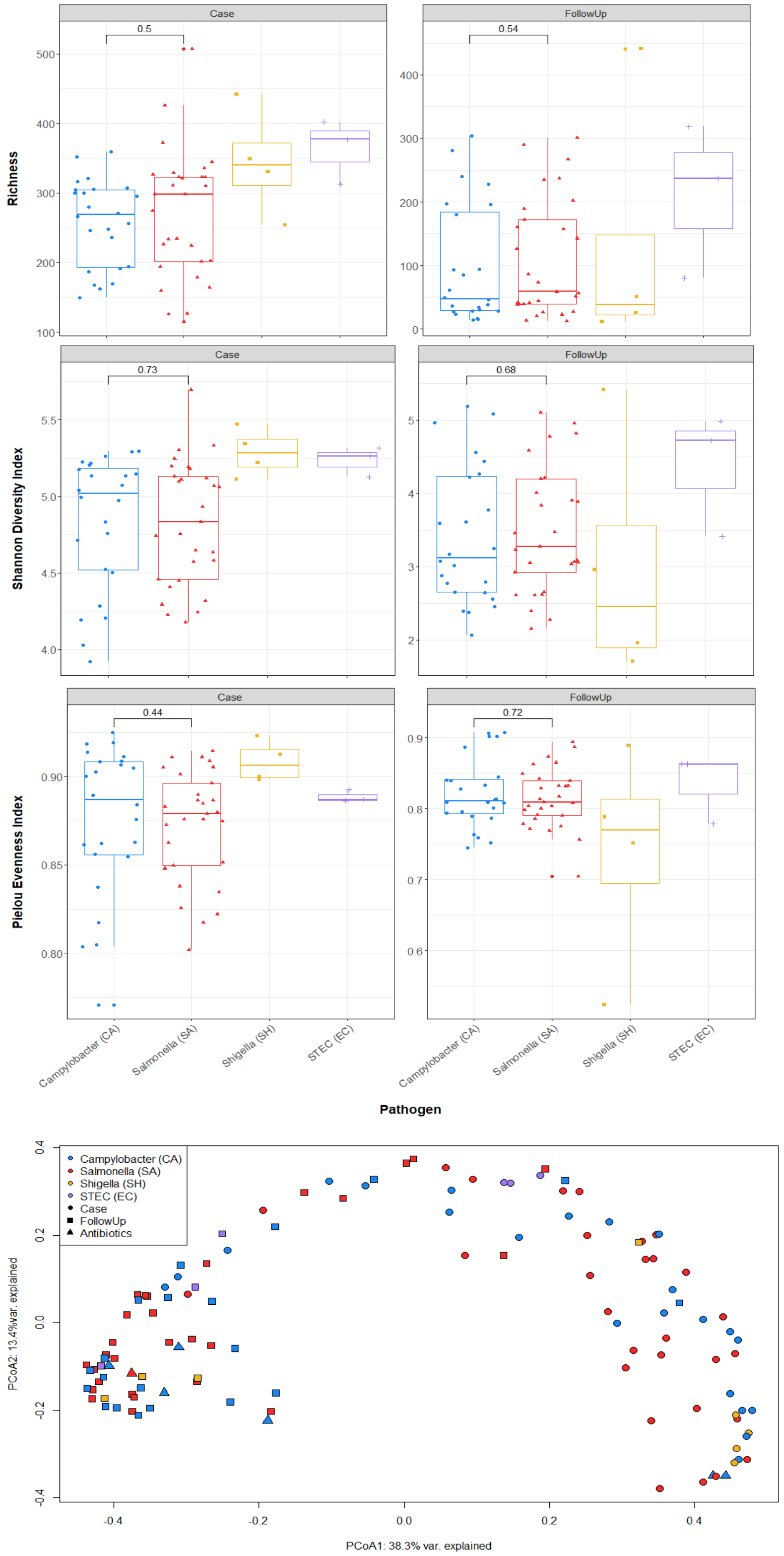

Figure S8. Alpha and beta diversity of the resistome do not differ across the four different enteric pathogens. Box plots for the three resistome alpha diversity measures (Richness, Shannon's Diversity Index, and Pielou's Evenness Index) are shown with separate plots for the case (Case, left) and follow-up (FollowUp, right) samples. Each plot is stratified by the pathogen

linked to the acute infection: *Campylobacter* (blue circles), *Salmonella* (red triangles), *Shigella* (yellow squares), and Shiga toxin-producing *Escherichia coli* (STEC; purple pluses). Points are offset from the vertical for clarity and the median is indicated by the thick bar. The first and third quartiles are represented by the bottom and top of the box, respectively. P- values comparing *Campylobacter* and *Salmonella* were calculated using the Wilcoxon rank-sum test and are shown above the comparison bar within each plot. Comparisons with S*higella* and STEC were not pursued because of low sample sizes. The bottom graph shows the Principal Coordinates Analysis (PCoA) plot based on Beta-diversity dissimilarity calculated from gene- level abundances for cases (circles) and follow-ups (squares). Patients reporting antibiotic use two weeks prior to sample collection are indicated by triangular data points. Colored points refer to the different pathogens: *Campylobacter* (blue), *Salmonella* (red), *Shigella* (yellow), STEC (purple). The first and second coordinate shows the corresponding percentage of similarity explained.

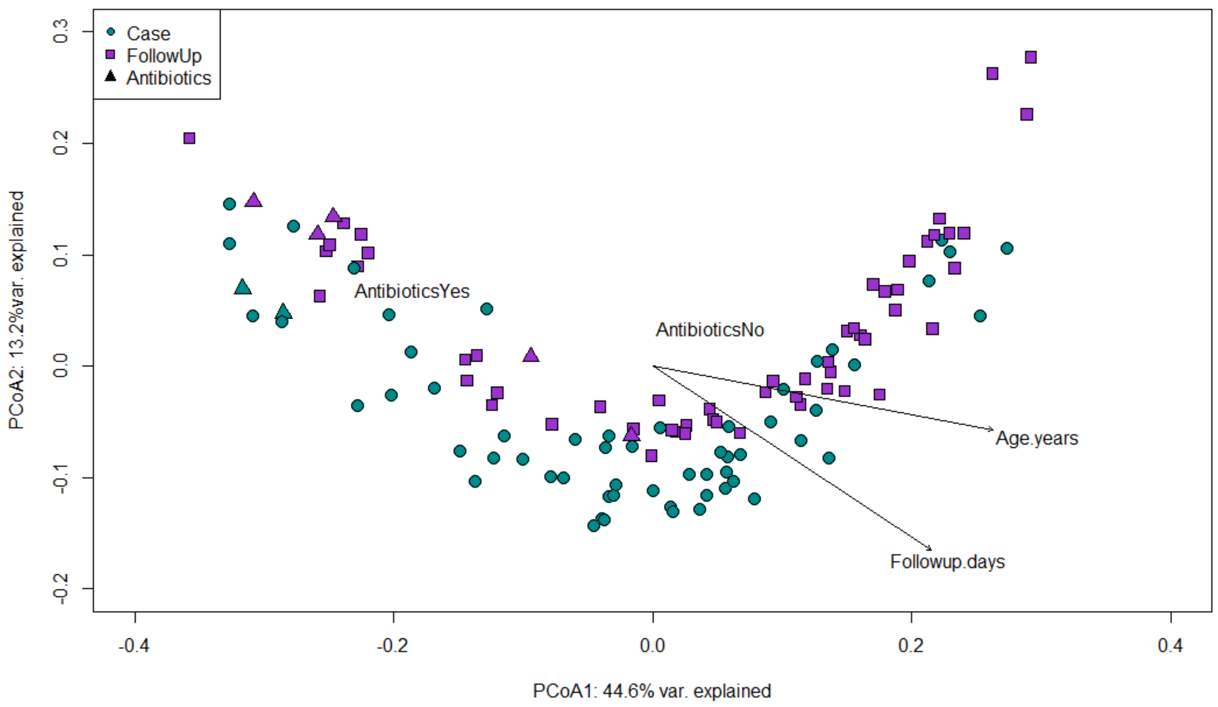

Figure S9. Certain variables drive the distribution of points among paired cases and follow-ups. A Principal Coordinates Analysis plot is shown for paired case (Case, green circles) and follow-up (FollowUp, purple squares) based on Bray-Curtis dissimilarity at the species level. The first and second coordinate are shown and include the corresponding percentage of similarity explained. Samples from individuals self-reporting use of antibiotics two weeks prior to sample collection are indicated triangular data points. A biplot was overlaid to display variables that had a significant influence on the distribution of points in the ordination. Age (Age.years), number of follow-up days (Followup.days), and antibiotic use (Yes and No) were influential vectors.

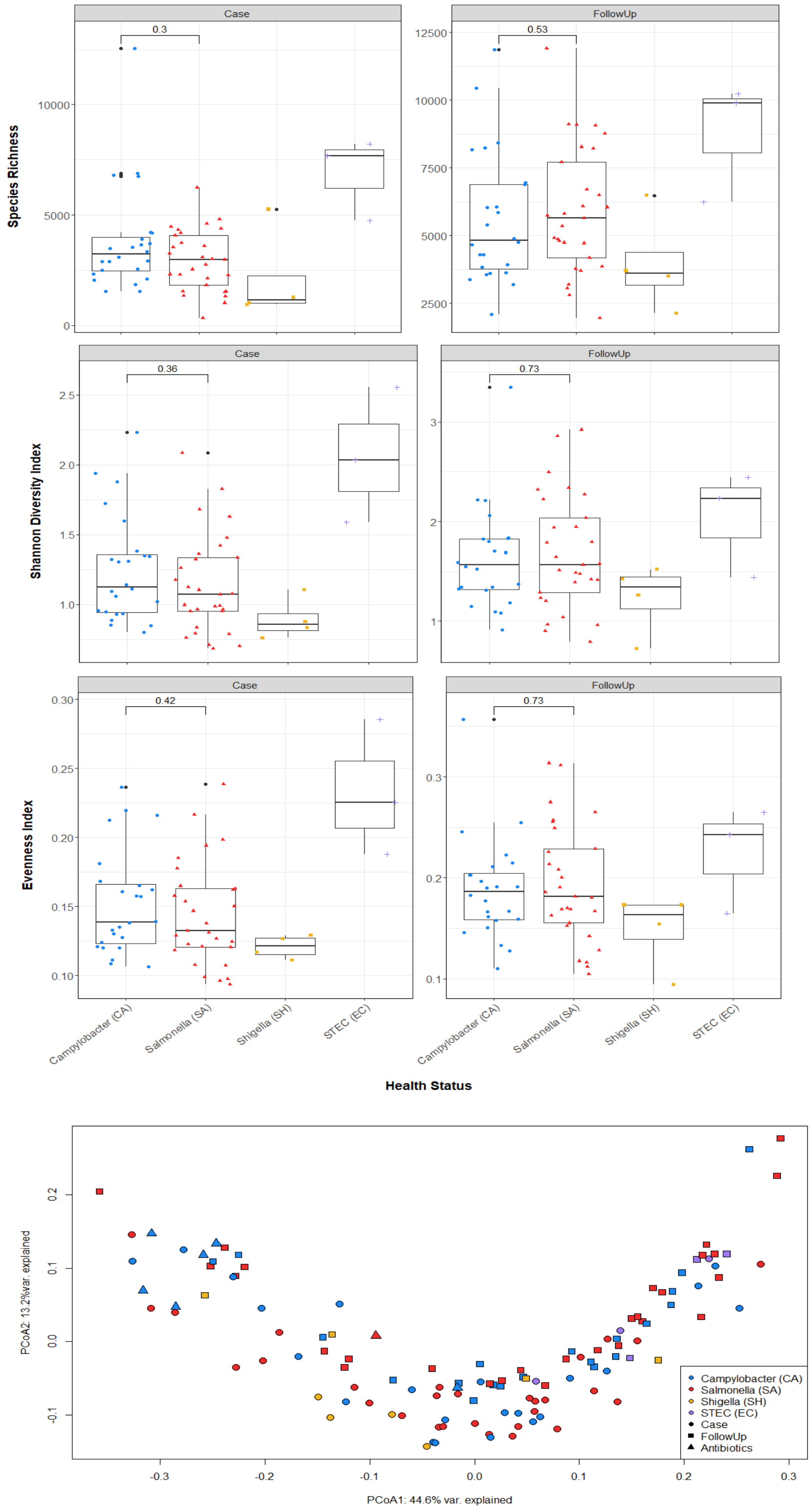

Figure S10. Microbiota diversity and composition do not differ across the four different enteric pathogens. Box plots for the three microbiome alpha diversity measures (Richness, Shannon's Diversity Index, and Pielou's Evenness Index) are shown with separate plots for the case

(Case, left) and follow-up (FollowUp, right) samples. Each plot is stratified by the pathogen linked to the acute infection: *Campylobacter* (blue circles), *Salmonella* (red triangles), *Shigella* (yellow squares), and Shiga toxin-producing *Escherichia coli* (STEC; purple pluses). Points are offset from the vertical for clarity and the median is indicated by the thick bar. The first and third quartiles are represented by the bottom and top of the box, respectively. P- values comparing *Campylobacter* and *Salmonella* were calculated using the Wilcoxon rank-sum test and are shown above the comparison bar within each plot. The bottom panel shows the Principal Coordinates Analysis plot based on Beta-diversity dissimilarity calculated from gene- level abundances for cases (circles) and follow-ups (squares). Points are coded for antibiotic use (triangles) and the four different pathogens: *Campylobacter* (blue), *Salmonella* (red), *Shigella* (yellow), STEC (purple). The first and second coordinate shows the corresponding percentage of similarity explained.

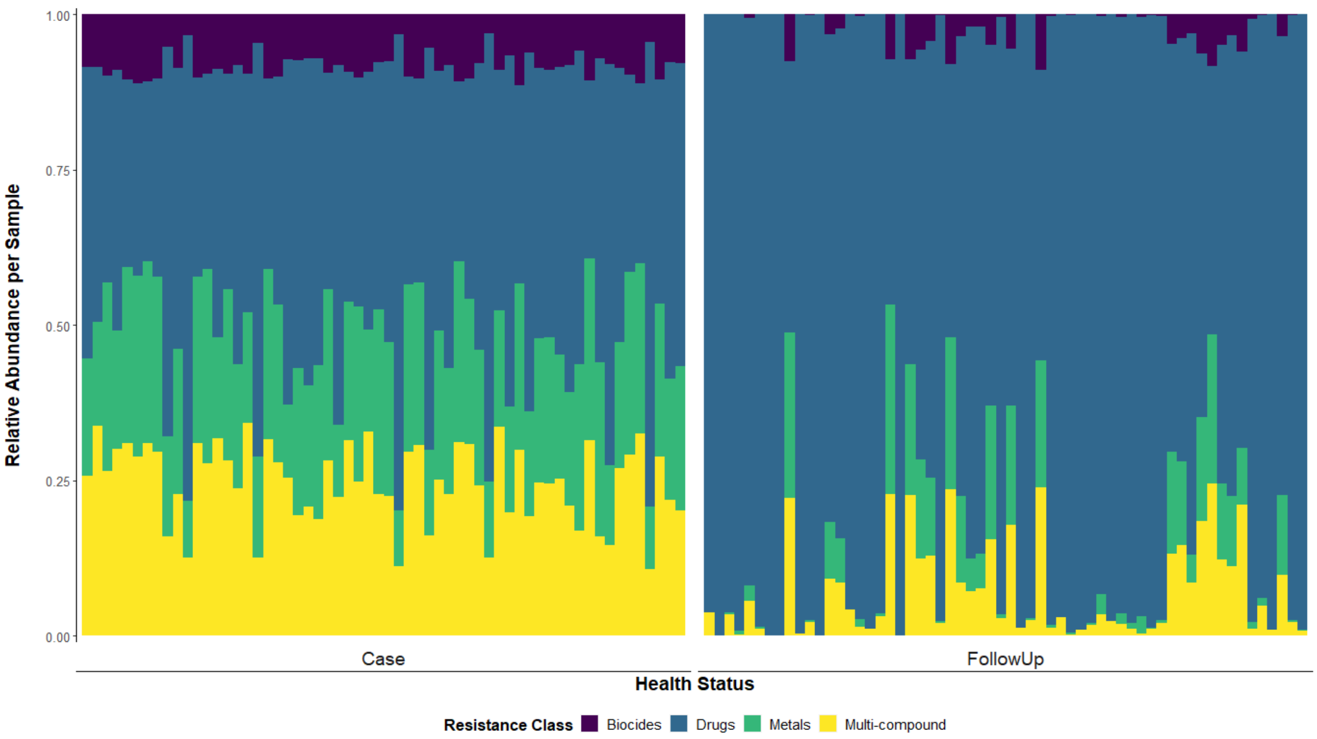

Figure S11. Relative abundance of resistance gene types in case and follow-up samples. The relative abundance of resistance genes assigned to biocides, drugs, metals, and multi-compounds is shown for cases (Case, left panel) and follow-ups (FollowUp, right panel), with each column representing the resistome from one individual. Columns are ordered by the paired sample, meaning that the column position in each side of the plot refers to the same individual either during or after enteric infection. Relative abundances were determined using raw gene abundances that had been normalized by the estimated number of genome equivalents in a given sample.

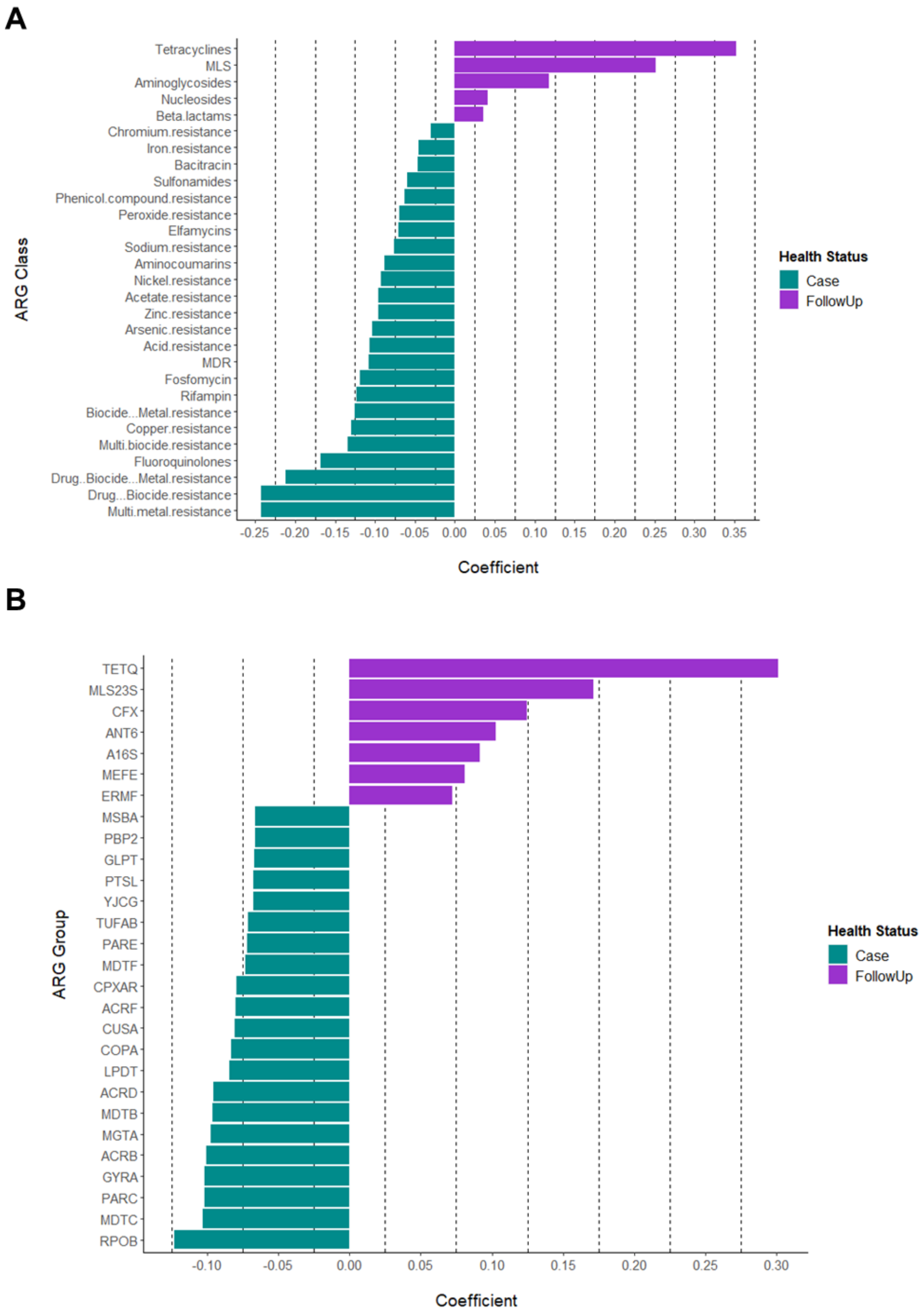

Figure S12. Differentially abundant ARG classes and groups among cases and follow-ups. MMUPHin was used to identify ARG features of differential abundance. Coefficients for each ARG class (A) or ARG group (B) are shown on the x-axis with a cutoff of absolute value = 0.05; positive coefficients indicate ARG features which were more abundant among follow-ups, while those with negative coefficients were more prominent in cases. The specific ARG features are displayed on the y-axis. The bars in the plot are colored by health status with which that particular feature is associated (cases=green, follow-ups=purple).

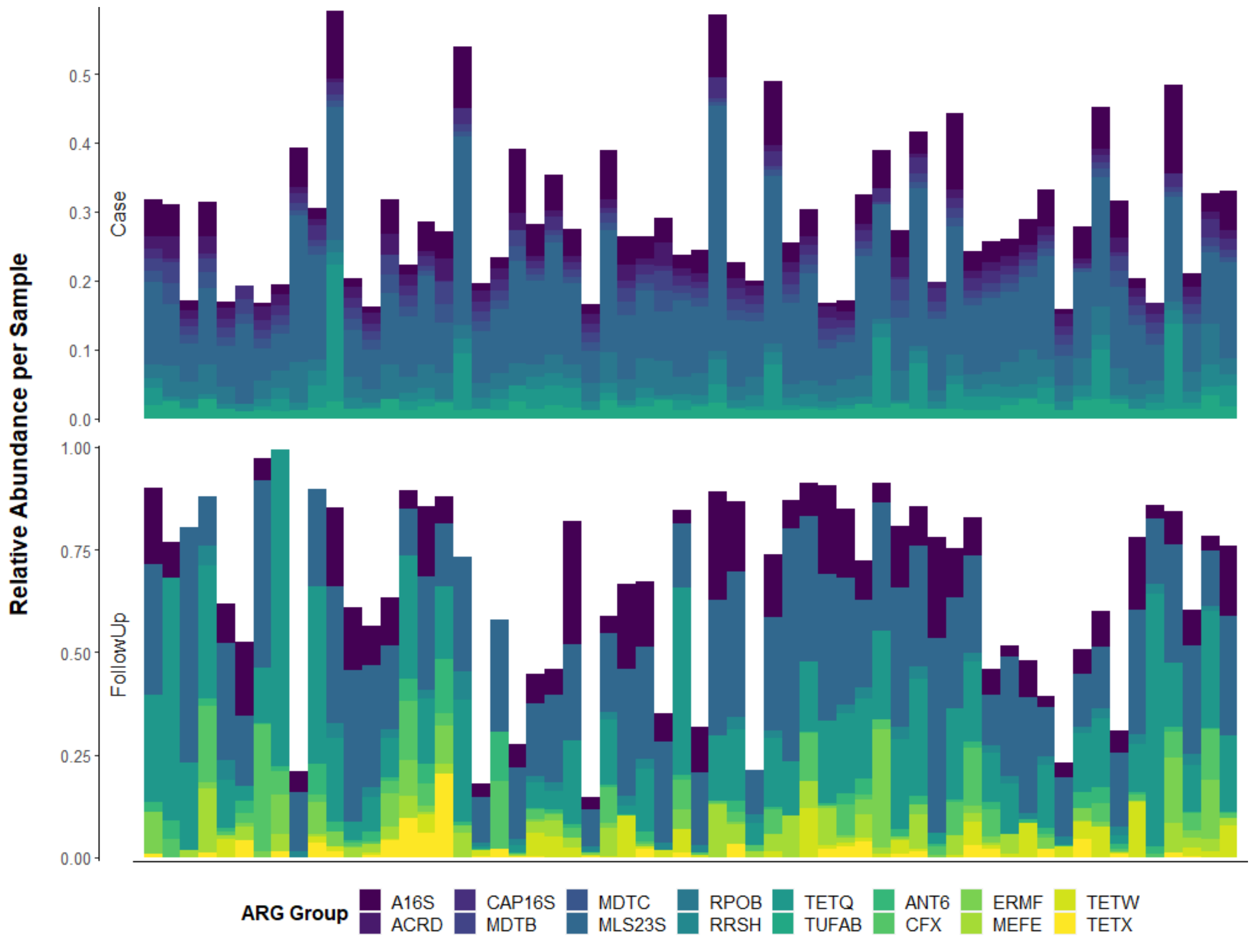

Figure S13. Relative abundance of the top-10 resistance gene groups among cases and follow-ups. The relative abundance of resistance genes assigned to the top-10 most abundant groups is shown for cases (Case, top panel) and follow-ups (FollowUp, bottom panel), with each column representing the resistome from one individual. Columns are ordered by individual, meaning that the column position in each side of the plot refers to the samples from the same individual during (top) or after (bottom) enteric infection. Relative abundances were determined using raw gene abundances that had been normalized by the estimated number of genome equivalents in a sample.

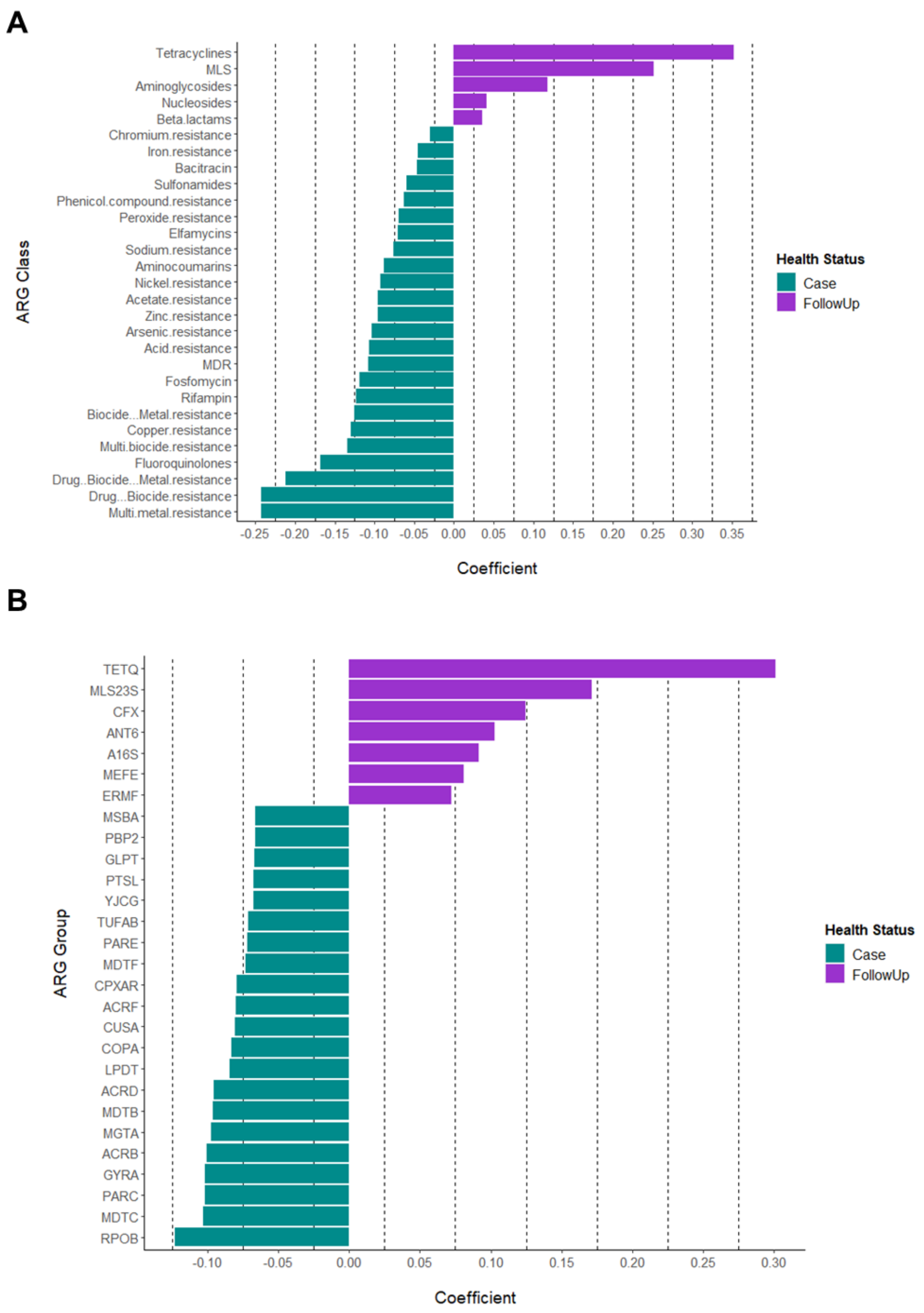

Figure S14. Differentially abundant ARG classes and groups among cases and follow-ups. MMUPHin was used to identify ARG features of differential abundance. Coefficients for each ARG class (A) or ARG group (B) are shown on the x-axis with a cutoff of absolute value = 0.05; positive coefficients indicate ARG features which were more abundant among follow-ups, while those with negative coefficients were more prominent in cases. The specific ARG features are displayed on the y-axis. The bars in the plot are colored by health status with which that particular feature is associated (cases=green, follow-ups=purple).

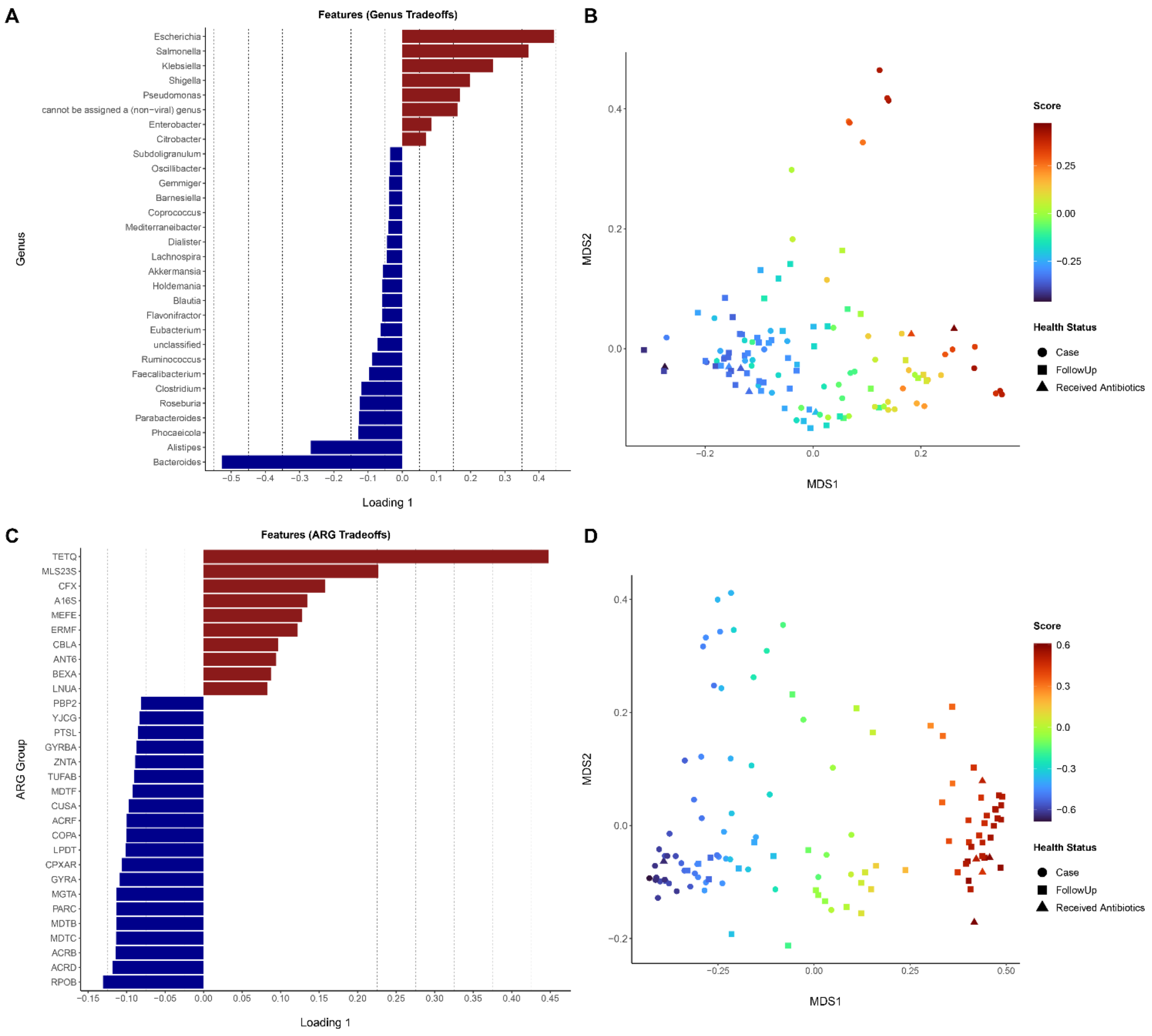

Figure S15. Continuous structure analysis reveals gradients driving the distribution of samples across the population. The top consensus loadings of the PCA for **A)** genus and **C)** antibiotic resistance gene (ARG) groups are shown stratified by sample for cases (Case=red) and follow-ups (FollowUp=blue) drawn from the differential abundance analyses. The composition gradients at the **B)** genus and **D)** ARG group levels overlaid onto ordination plots based on Bray-Curtis dissimilarity. Cases (circles), follow-ups (squares), and individuals who received antibiotics (triangles) are shown. The color gradient ("Score") refers to the continuous structure score affiliated with Loading 1 for phyla and genera, respectively. Juxtaposition of (A-C) and (B-D) allow interpretation of tradeoffs within the samples.

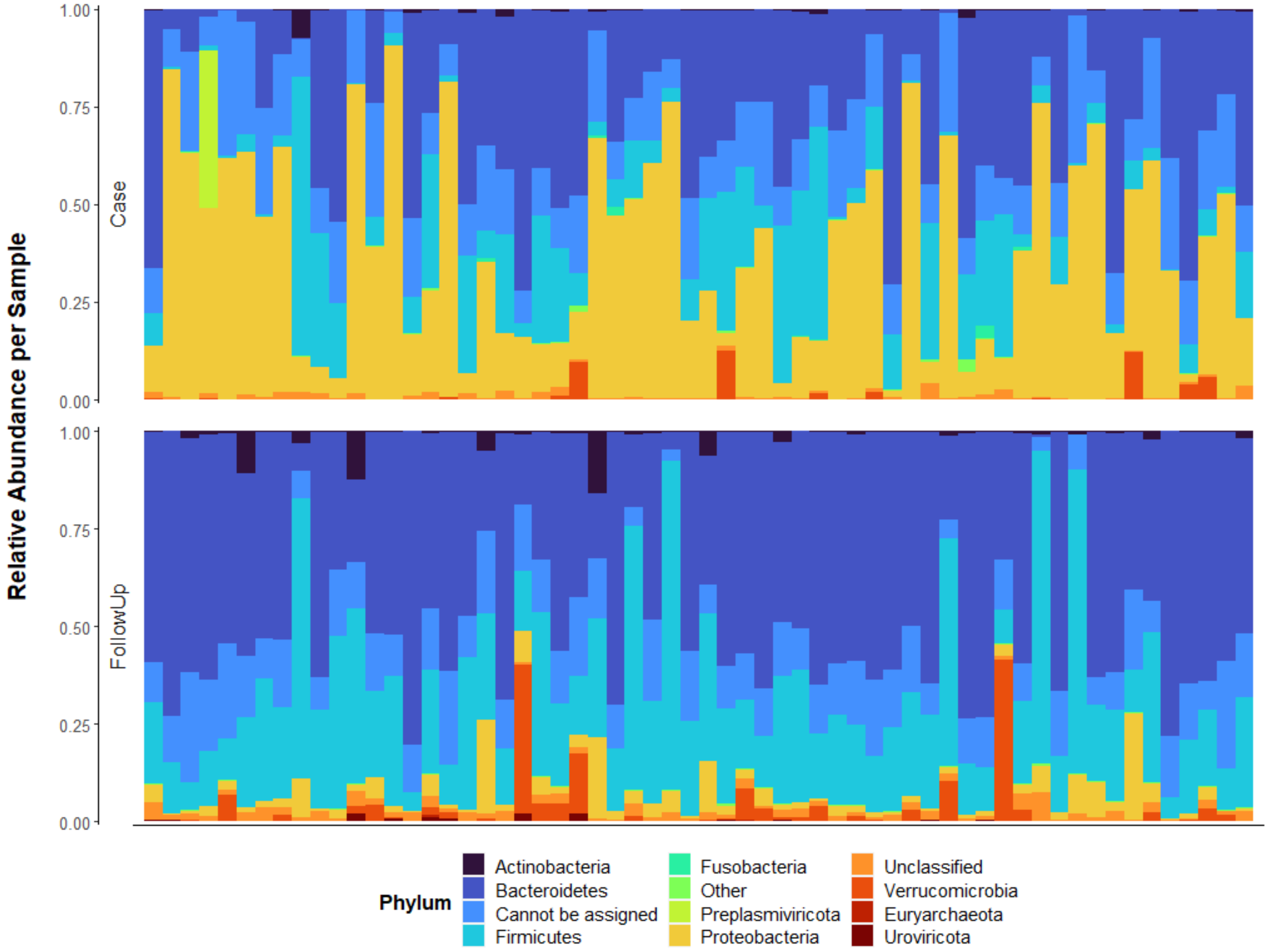

Figure S16. Relative abundance of microbial phyla differ between cases and follow- ups. The top-10 microbial phyla with the greatest average relative abundance among cases or follow-ups is shown with each column representing the microbiome from one individual. Columns are ordered by their sample pairing, meaning that the column position for each facet of the plot refers to the same individual either during (Case; Top) or after (FollowUp; Bottom) enteric infection. Relative abundances were determined using raw gene abundances that had been normalized by the approximate number of genome equivalents in the sample as determined using MicrobeCensus.

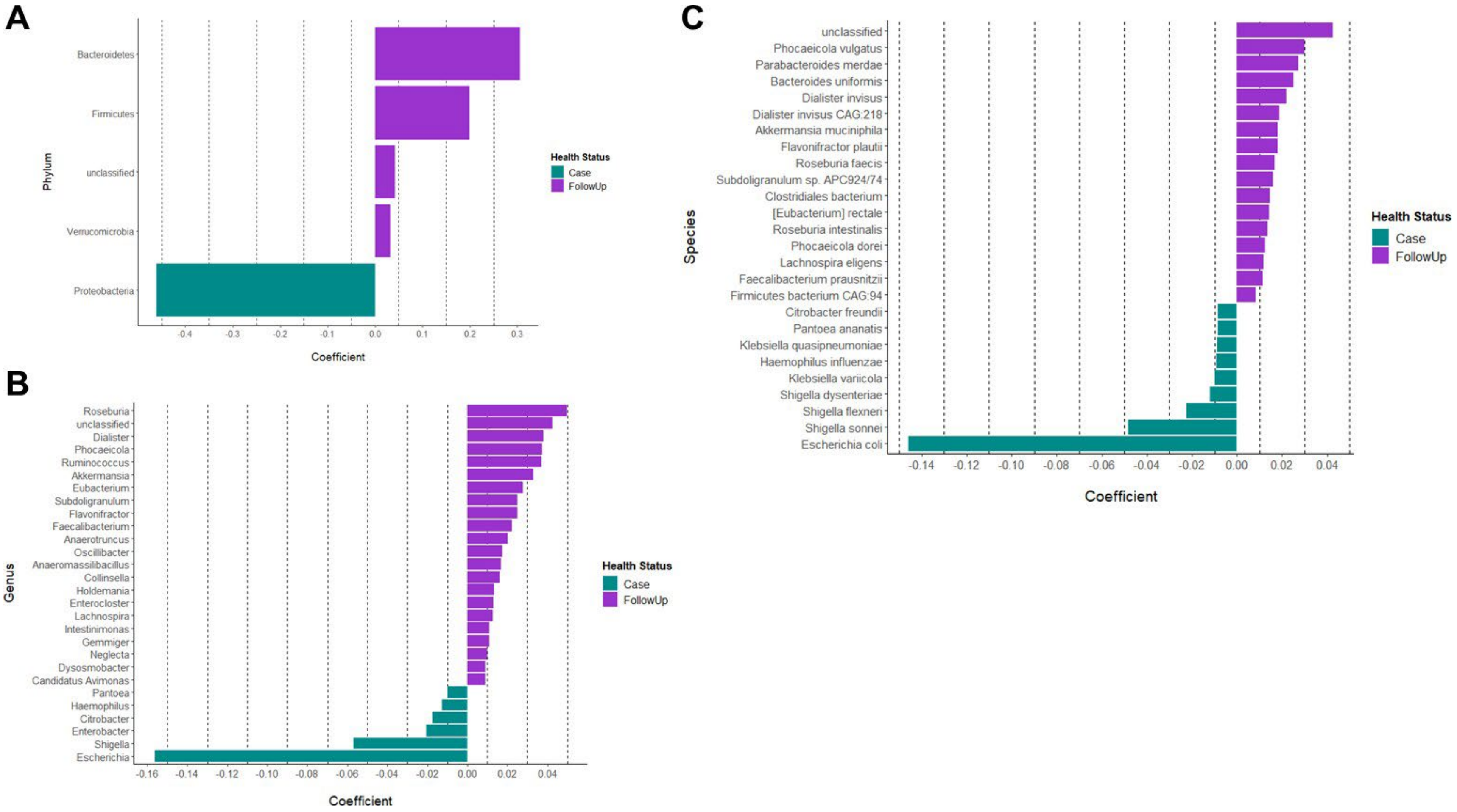

Figure S17. Differential abundance of phyla, genera, and species among cases and follow-ups. MMUPHin was used to identify microbial features of differential abundance. Coefficients for each phylum (A) genus (B) or species (C) are shown on the x- axis with a cutoff of absolute value=0.05, 0.008, and 0.008, respectively; positive coefficients indicate features that were more abundant among follow-ups, while negative coefficients indicate those that were more abundant cases. The specific taxonomic features are displayed on the y-axis. Bars in the plot are colored by sample (cases=green, follow-ups=purple).

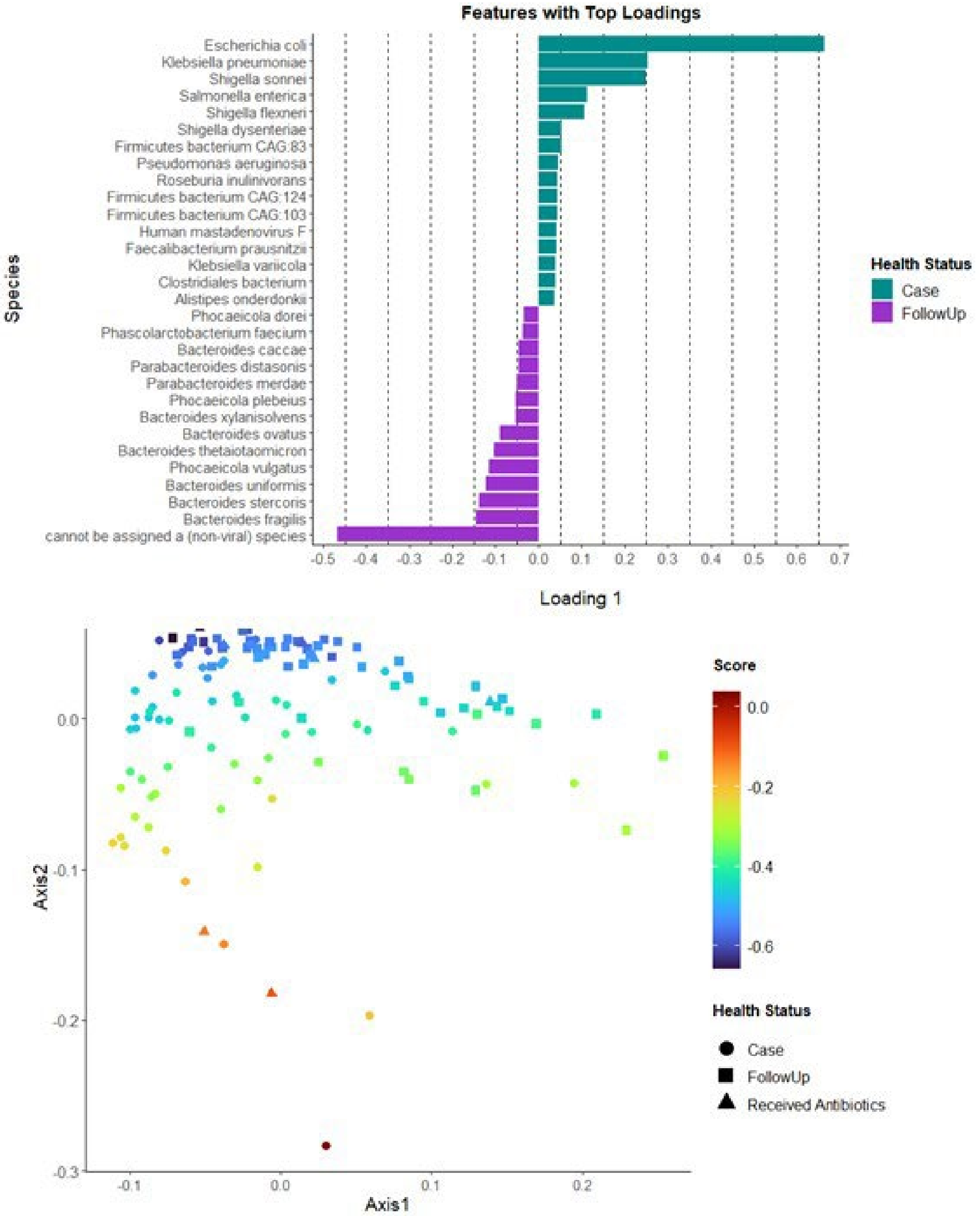

Figure S18. Continuous structure analysis reveals species gradients among cases and follow-ups. MMUPHin was used to investigate potential continuous structure among the microbiome composition of cases and follow-ups at the species level. **A)** Species determined to comprise the top consensus loadings are shown; colors have been assigned to the loadings based on the sample affiliated with each loading (drawn from differential abundance analyses). Cases (Case) are indicated in green and follow-ups (FollowUp) in purple. **B)** The species composition gradient is shown overlaid onto an ordination plot based on Bray-Curtis dissimilarity of case and follow-up microbiomes at the species level. Cases (circles), follow-ups (squares), and individuals who received antibiotics (triangles) are shown. The color gradient ("Score") refers to the continuous structure score affiliated with Loading 1. Juxtaposition of (A) and (B) allow interpretation of species tradeoffs that occur within the sample set (e.g., many cases contain higher levels of *Escherichia coli* at the expense of more beneficial bacteria such as Bacteroides species associated with the opposite direction.

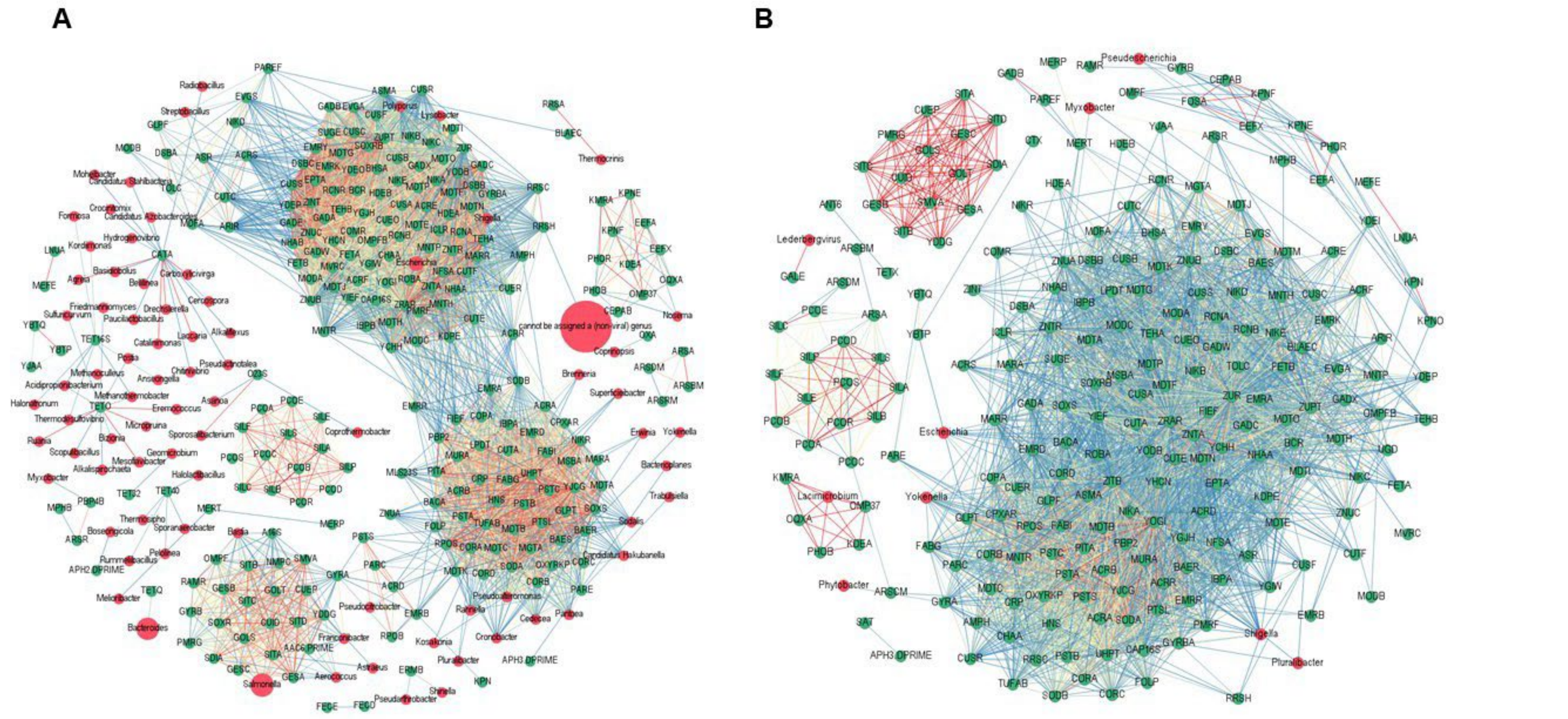

Figure S19. **ARG-ARG and ARG-taxa connections are different for individuals infected with and recovering from *Salmonella*.** Correlation co-occurrence networks were constructed in Gephi 0.9.2 using Spearman's Rank correlation coefficients generated with the R-package 'Hmisc' (v4.5-0) for cases infected with *Salmonella* **(A)** or recovering (follow-ups) **(B)**. These networks display all ARG-ARG and ARG-taxa connections; taxa-taxa connections were excluded for clarity. Correlations included in the network all passed a cutoff of $\rho>0.80$ and q-value < 0.05. Nodes are colored by their identity as a taxonomic genus (red) or ARG group (green). Nodes are sized based on their overall abundance among samples; the larger the node, the more abundant. Nodes with ≥ 1 connection were included (i.e. degree cutoff=1). The edge color displays the strength of correlation, with blue demonstrating relatively weaker correlations (yet still >0.80), yellow showing medium correlation, and red showing strong correlation. Nodes are labeled with their corresponding genus or ARG group.

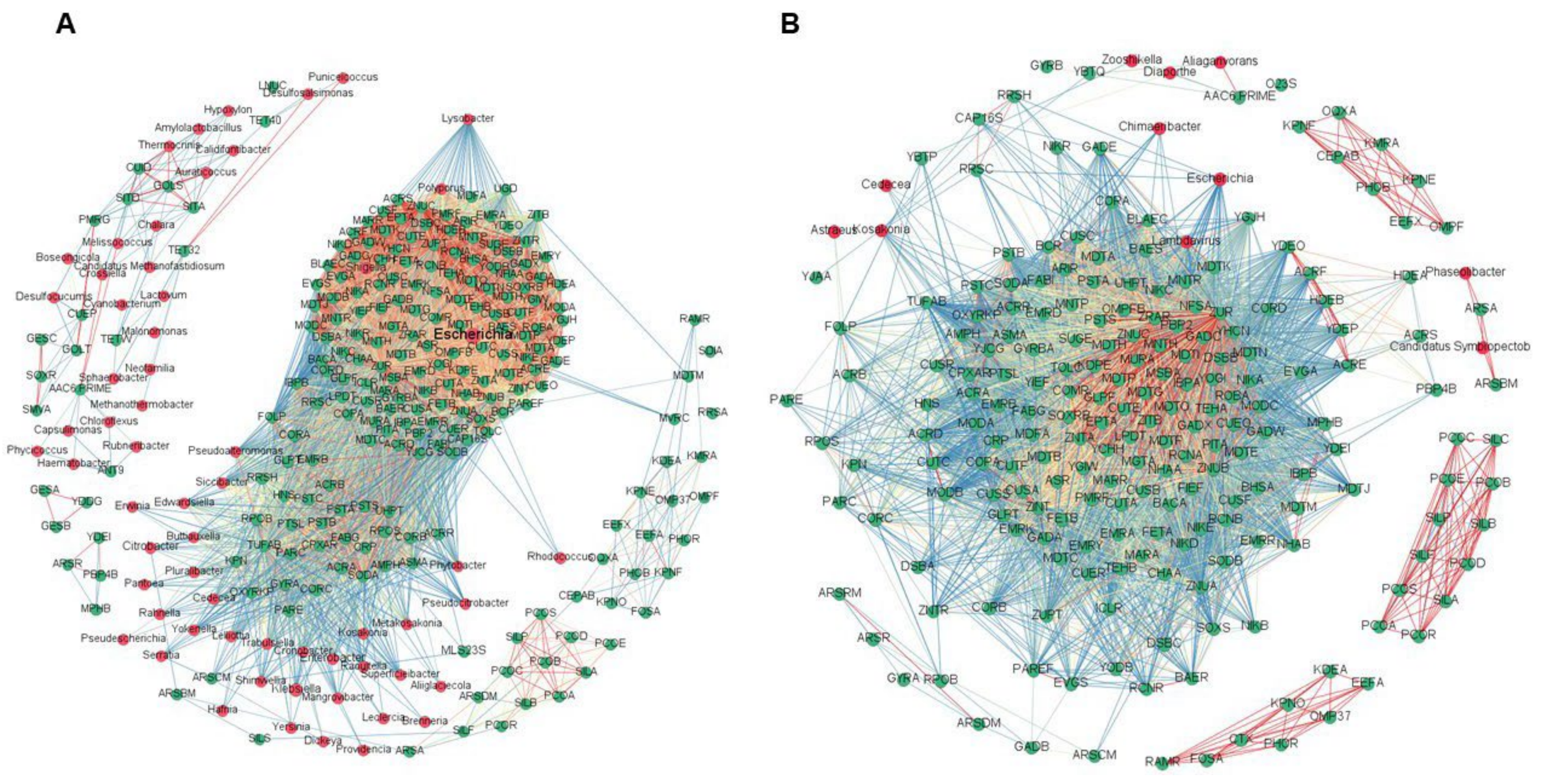

Figure S20. ARG-ARG and ARG-taxa connections are different for individuals infected with and recovering from *Campylobacter*. Correlation co-occurrence networks were constructed in Gephi 0.9.2 using Spearman's Rank correlation coefficients generated with the R-package 'Hmisc' (v4.5-0) for cases infected with *Campylobacter* (A) and recovering follow-ups (B). These networks display all ARG-ARG and ARG-taxa connections; taxa-taxa connections were excluded for clarity. Correlations included in the network all passed a cutoff of $\rho > 0.80$ and q-value < 0.05. Nodes are colored by their identity as a taxonomic genus (red) or ARG group (green). Nodes are sized based on their overall abundance among samples; the larger the node, the more abundant. Nodes with $\geq$ 2 connections were included (i.e. degree cutoff=2). The edge color displays the strength of correlation, with blue demonstrating relatively weaker correlations (yet still >0.80), yellow showing medium correlation, and red showing strong correlation. Nodes are labeled with their corresponding genus or ARG group.

Table S1. Shapiro-Wilk Test results to determine whether the data for case and follow-up samples are normally distributed in the microbiome and resistome datasets.

Dataset	Alpha Diversity Metric	Test Statistic (W)	p-value
Microbiome	Richness	0.936	2.35E-05
	Shannon Diversity	0.933	1.45E-05
	Pielou's Evenness	0.940	4.25E-05
Resistome	Richness	0.940	4.18E-05
	Shannon Diversity	0.905	3.63E-07
	Pielou's Evenness	0.885	3.68E-08

Table S2. Summary of beta-lactamase genes and their corresponding microbial hosts in cases and follow-ups. The number of resistance genes in cases vs. follow-ups is shown and genes encoding extended-spectrum beta-lactamase (ESBL) production are indicted with an asterisk (*).

Microbial Host	Beta-lactamase	Cases	Follow-ups
Enterobacter	ACT family cephalosporin-hydrolyzing class C beta-lactamase	5	0
Acinetobacter	ADC family extended-spectrum class C beta-lactamase*	2	0
Klebsiella	Beta-lactamase	0	1
**Uncultured** [a]	beta-lactamase	0	1
Klebsiella	beta-lactamase	4	0
Escherichia	beta-lactamase	3	6
Escherichia	beta-lactamase TEM-1 variant*	1	0
**Synthetic** [b]	beta-lactamase TEM-1 variant*	19	4
Klebsiella	beta-lactamase, partial	3	0
Shigella	BlaEC family class C beta-lactamase	1	0
Escherichia	BlaEC family class C beta-lactamase	49	19
Bacteroides	CepA family class A extended-spectrum beta-lactamase*	19	13
Prevotella	CfxA family class A broad-spectrum beta-lactamase	5	9
Bacteroides	CfxA family class A broad-spectrum beta-lactamase	46	48
Atlantibacter	class A beta-lactamase	1	0
Burkholderia	class A beta-lactamase	0	1
Proteus	class A beta-lactamase	1	0
Salmonella	class A beta-lactamase	1	0
Yersinia	class A beta-lactamase	0	1
Bacillus	class A beta-lactamase	1	1
Clostridium	class A beta-lactamase	1	3
Klebsiella	class A beta-lactamase, partial	1	0
Escherichia	class A beta-lactamase, partial	0	2
Bacteroides	class A beta-lactamase, subclass A2	45	47
Providencia	class C beta-lactamase	1	0
Hafnia	class C beta-lactamase	3	0
Pseudomonas	class C beta-lactamase	7	0
Flavobacterium	class D beta-lactamase	4	6

Microbial Host	Beta-lactamase	Cases	Follow-ups
Klebsiella	class D beta-lactamase, partial	1	0
Salmonella	CMY family class C beta-lactamase	3	2
Salmonella	CMY-2 family class C beta-lactamase	2	1
Citrobacter	CMY-2 family class C beta-lactamase	8	3
Morganella	DHA family class C beta-lactamase	1	0
Klebsiella	LEN family class A beta-lactamase	1	2
Enterobacter	MIR family cephalosporin-hydrolyzing class C beta-lactamase	1	0
Klebsiella	MULTISPECIES: OXY family class A extended-spectrum beta-lactamase*	2	0
Klebsiella	OXA-1 family class D beta-lactamase*	2	0
Pseudomonas	OXA-50 family oxacillin-hydrolyzing class D beta-lactamase*	2	0
Acinetobacter	OXA-51 family carbapenem-hydrolyzing class D beta-lactamase*	2	0
Campylobacter	OXA-61 family class D beta-lactamase*	2	0
Klebsiella	OXY family class A extended-spectrum beta-lactamase*	2	0
Pseudomonas	PDC family class C beta-lactamase	2	0
Burkholderia	PenA family class A beta-lactamase	1	0
Raoultella	PLA/ORN/TER family class A beta-lactamase	2	0
Citrobacter	SED family class A beta-lactamase	4	3
Klebsiella	SHV family class A beta-lactamase*	8	2
Desulfovibrio	subclass B1 metallo-beta-lactamase	0	1
Myxococcus	subclass B1 metallo-beta-lactamase	1	0
Bacteroides	subclass B1 metallo-beta-lactamase	8	14

[a] "Uncultured" indicates that the taxon identified to carry the associate beta-lactamase has yet to be cultured, sequenced, and identified.
[b] "Synthetic" indicates that the BLASTP search identified an ESBL in a synthetic gene sequence, which may suggest low resolution for this particular ACC and an inability to assign a taxon.
